# MBD2 regulates the progression and chemoresistance of cholangiocarcinoma through interaction with WDR5

**DOI:** 10.1186/s13046-024-03188-4

**Published:** 2024-09-30

**Authors:** Da Wang, Junsheng Chen, Guanhua Wu, Fei Xiong, Wenzheng Liu, Qi Wang, Yiyang Kuai, Wenhua Huang, Yongqiang Qi, Bing Wang, Ruizhi He, Yongjun Chen

**Affiliations:** 1 Department of Biliary-Pancreatic Surgery, Tongji Hospital, Tongji Medical College, Huazhong University of Science and Technology, 1095 Jiefang Avenue, Wuhan, Hubei 430074 China; 2 Department of General Surgery, Beijing Friendship Hospital, Capital Medical University, Beijing, 100050 China; 3 Department of Emergency, Tongji Hospital, Tongji Medical College, Huazhong University of Science and Technology, 1095 Jiefang Avenue, Wuhan, 430074 Hubei China; 4 Key Laboratory of Laparoscopic Technology of Zhejiang Province, Department of General Surgery, Sir Run- Run Shaw Hospital, Zhejiang University School of Medicine, Hangzhou, 310016 China

**Keywords:** MBD2, WDR5, ABCB1, H3K4me3, Cholangiocarcinoma

## Abstract

**Background:**

Cholangiocarcinoma (CCA) is a highly malignant, rapidly progressing tumor of the bile duct. Owing to its chemoresistance, it always has an extremely poor prognosis. Therefore, detailed elucidation of the mechanisms of chemoresistance and identification of therapeutic targets are still needed.

**Methods:**

We analyzed the expression of MBD2 (Methyl-CpG-binding domain 2) in CCA and normal bile duct tissues using the public database and immunohistochemistry (IHC). The roles of MBD2 in CCA cell proliferation, migration, and chemoresistance ability were validated through CCK-8, plate cloning assay, wound healing assays and xenograft mouse model. In addition, we constructed a primary CCA mouse model to further confirm the effect of MBD2. RNA-seq and co-IP-MS were used to identify the mechanisms by how MBD2 leads to chemoresistance.

**Results:**

MBD2 was upregulated in CCA. It promoted the proliferation, migration and chemoresistance of CCA cells. Mechanistically, MBD2 directly interacted with WDR5, bound to the promoter of ABCB1, promoted the trimethylation of H3K4 in this region through KMT2A, and activated the expression of ABCB1. Knocking down WDR5 or KMT2A blocked the transcriptional activation of ABCB1 by MBD2. The molecular inhibitor MM-102 targeted the interaction of WDR5 with KMT2A. MM-102 inhibited the expression of ABCB1 in CCA cells and decreased the chemoresistance of CCA to cisplatin.

**Conclusion:**

MBD2 promotes the progression and chemoresistance of CCA through interactions with WDR5. MM-102 can effectively block this process and increase the sensitivity of CCA to cisplatin.

**Supplementary Information:**

The online version contains supplementary material available at 10.1186/s13046-024-03188-4.

## Background

Cholangiocarcinoma (CCA) is a highly lethal malignant tumor originating from the epithelium of the biliary tree [1]. The morbidity of CCA has increased worldwide in recent years [2]. CCA has no obvious clinical symptoms in the early stage. Symptoms such as jaundice and abdominal pain are apparent until the advanced stage, at which point most patients have lost the opportunity for surgery [3]. The combination of gemcitabine and cisplatin is considered the standard chemotherapy regimen for the treatment of CCA but improves the median survival by only 1 year [4, 5]. Given the above findings, identifying a therapeutic target, especially a chemoresistance target, is particularly urgent.

MBD2 (methyl-CpG-binding domain 2) is an MBD protein that functions as a DNA methylation ‘reader’ to specifically bind methylated CpG dinucleotides and regulate transcription processes [6]. MBD2 contains an MBD domain, transcriptional repression domain (TRD), C-terminal coiled coil (CC) domain and glycine‒arginine (GR) repeat region. The MBD domain can bind methylated CpG dinucleotides. The TRD and CC domains are mainly responsible for protein‒protein interactions, and the GR repeat region is subjected to posttranslational modifications [7, 8]. MBD2 can recruit histone deacetylases (NuRDs) to methylated CpG dinucleotide regions of genes, reduce histone acetylation levels, and lead to transcriptional silencing. However, the functions of MBD2 are not all related to silencing. Previous studies using ChIP-seq data have shown that MBD2 can bind to transcriptionally active promoters, which indicates that MBD2 may have a transcriptional activation effect [9, 10]; however, its mechanisms are less understood. Researchers have suggested that the transcriptional activation by MBD2 may be due to its interactions with other proteins instead of NuRD at specific loci. MBD2/TACC3 form a complex with pCAF and bind at gene promoters to reactivate transcription [11]. The above research indicates that the mechanism of MBD2 is variable and complex, and further in-depth research is needed. MBD2 has been reported to promote tumor progression in acute myeloid leukemia (AML) [12], colorectal cancer [13], and medulloblastomas [14], but less research has been conducted in CCA.

The WD repeat domain 5 (WDR5) protein, an essential component of H3K4 methyltransferase complexes, consists of 7 typical WD40 repeat domains that interact with the N-terminal end of the histone H3 tail. Moreover, WDR5 interacts with MLL, RBBP5, ASH2L, and DPY30 to form a complex that catalyzes the trimethylation of H3K4 [15]. Notably, the absence of WDR5 results in complete loss of H3K4 methylation, whereas the absence of RBBP5, ASH2L or DPY30 results in partial loss of H3K4 methylation [16]. WDR5 connects H3 and MLL, playing an indispensable role in this complex. WDR5 recruits MLL to the promoters of leukemia-related genes, leading to the activation of these oncogenes and promoting MLL-rearranged leukemia [17]. In addition, WDR5 plays key roles in the progression of a variety of cancers, such as pancreatic cancer [18], breast cancer [19], and neuroblastoma [20]. Moreover, the TOX3–WDR5 complex accelerates the chemoresistance of colorectal cancer [21]. Therefore, WDR5 is expected to become an effective therapeutic target for cancer.

The use of antitumor drugs has become a routine method for treating cancer, especially CCA [4]. However, the development of drug resistance makes cancer cells insensitive to these drugs, which has become an important factor impacting treatment efficacy. ATP-binding cassette transporter B1 (ABCB1) is a member of the ABC protein family and is responsible for multidrug resistance (MDR) through excreting drugs from cells [22]. The binding of the substrate induces a conformational change in ABCB1, removing the substrate from the cell via the hydrolysis of ATP. Although some progress has been made in the combination of ABCB1 inhibitors and chemotherapy drugs, owing to their significant side effects, no drugs have been approved for clinical sensitization therapy [23]. Therefore, identification of the upstream regulatory mechanism of ABCB1 and exploration of new therapeutic targets are urgently needed.

In this study, we provide evidence that MBD2 can upregulate the expression of ABCB1 and promote the chemoresistance, proliferation and migration abilities of CCA. Mechanistically, WDR5 was found to be recruited by MBD2 to the promoter of ABCB1, where it activated transcription through trimethylation of H3K4 by KMT2A. The WDR5–KMT2A inhibitor MM-102 effectively increased the sensitivity of CCA to cisplatin. Our research suggests that the MBD2-WDR5-KMT2A/ABCB1 signaling axis may be a potential target for the treatment of chemoresistance, proliferation, and migration in CCA.

## Methods

### Patient samples

The clinical characteristics and CCA tissues of forty patients with CCA were collected from June 2018 to October 2022 at Tongji Hospital, Huazhong University of Science and Technology (Wuhan, China). All of the patients were followed after discharge, with follow-up data collected up to May 2024. This research was reviewed and approved by the Ethics Committee of Tongji Hospital of Huazhong University of Science and Technology.

### Cell culture, lentivirus infection and plasmid transfection

The human CCA cell lines TFK1 and RBE were cultured in RPMI 1640 supplemented with 10% fetal bovine serum (FBS). Human embryonic kidney (HEK293T) cells were cultured in DMEM supplemented with 10% FBS. All of the cells were placed in a humidified incubator containing 5% CO_2_ at 37 °C. MM-102 (S7265; Selleck) was added to the cells at a concentration of 50 µM. Cisplatin was purchased from Selleck (S1166; Selleck).

Lentiviruses for knocking down or overexpressing target genes were produced by cotransfecting HEK293T cells with target plasmids and the packaging plasmids psPAX and pMD2G. The culture supernatant was collected after 72 h and filtered through a 0.45 μm membrane (BS-PES-45; Biosharp). TFK1 and RBE cells were transfected with 1 mL of lentivirus supernatant and cultured in 1 mL of complete RPMI 1640 medium and 2 µL of polybrene (40804ES76; Yeasen) for 24 h. After 72 h, puromycin (CL13900; Selleck, 1 µg/mL) or G-418 disulfate (HY-17561; MCE, 1 µg/mL) was added to screen positive cells for 10 days. Western blotting was performed to validate the efficiency of overexpression or knockdown.

### Western blot and antibodies

The cells were lysed in RIPA lysis buffer (50 mM Tris–HCl, 150 mM NaCl, 1 mM EDTA-Na2, 1% Triton X-100, 1% sodium deoxycholate, 0.1% SDS) supplemented with protease inhibitor cocktail (20124ES03; Yeasen). The total protein was heat-denatured with 5× loading buffer (G2013; Servicebio) at 95 °C for 10 min. Prepared samples with equal amounts of total protein (40 µg) were used for immunoblotting according to standard procedures. The antibodies used in this research are listed in Table S1.

### Co-immunoprecipitation

The cells were transfected with the corresponding plasmids, collected and lysed in IP buffer supplemented with complete protease inhibitor cocktail (20124ES03; Yeasen). The cell lysates were subjected to ultrasonication and centrifugation. Then, 40 µl of cell lysate was used as the input, and the rest of the cell lysate was incubated with A/G magnetic beads (B23202, Biomake, US) + the corresponding antibody or Flag-coupled magnetic beads (B26102, Biomake, US) at 4 °C for 12 h. The magnetic beads were subsequently washed five times for 5 min each wash with precooled IP buffer, and 40 µl of 2× loading buffer was added and incubated at 95 °C for 15 min. Immunoblotting and subsequent analysis were subsequently performed. The antibodies used are listed in Table S1.

### GST pull-down assay

The CDS regions of MBD2/WDR5 were subsequently cloned and inserted into the pGEX-4T-1 vector by PCR. The GST-Empty/GST-MBD2/GST-WDR5 plasmids were transfected into *E. coli* BL21 cells, and IPTG (final concentration 500 µM) was added at 16 °C for 12 h. Protease inhibitor cocktail addition buffer was added to the *E. coli*. The *E. coli* cells were then ultrasonically broken and centrifuged, the lysate was collected, and anti-GST magnetic beads (HY-K0222, MCE) were incubated on a rotating device at 4 °C for 1 h. The GST magnetic beads obtained in the above step were incubated with the cell lysates for 4 h at 4 °C on a rotating device. The magnetic beads were subsequently washed five times for 5 min each with precooled IP buffer, and 40 µl of 2× loading buffer was added and incubated at 95 °C for 15 min. Immunoblotting and subsequent analysis were then performed.

### Plasmid construction

The CDSs of the target genes were subsequently cloned and inserted into the Phage-Flag vector, Phage-HA vector or Phage vector. The shRNA primers were inserted into the plko.1 vector.

### Cell proliferation, plate colony formation and wound healing assays

For the cell proliferation assay, different groups of cells (500 cells/well) were cultured in 96-well plates with RPMI 1640 medium. Then, CCK-8 reagent (40203ES92; Yeasen) was added according to the manufacturer’s protocol. The cells were incubated at 37 °C and 5% CO_2_ for 90 min, after which the absorbance was measured at 450 nm. When CCK-8 was used to detect the sensitivity of CCA cells to cisplatin, the cell count was controlled at 2000 cells per well. After cell adhesion, the culture medium containing different concentrations of cisplatin was replaced, and after 48 h of exposure, cell activity was detected using CCK-8 reagent. Prism 8.0 was used to calculate the IC50 value.

For the plate colony formation, 500 cells were cultured in 3-cm dishes for 2 weeks, fixed with 4% formaldehyde for 15 min, and then stained with crystal violet for 15 min. Colonies with diameters greater than 100 μm were counted.

For the wound healing assay, 100% density CCA cells were seeded in 3-cm dishes, and after the cells had adhered to the walls, a pipette was used to draw lines inside the well. The culture medium was replaced with medium without FBS, and photos were taken every 48 h to observe the degree of cell migration. ImageJ was used to analyze the cell migration rate.

### ChIP‒qPCR

The CCA cell samples were fixed with 1% formaldehyde, and then lysis buffer was added to obtain the nuclear products. Ultrasonication was performed to fragment the nuclear products, and the samples were precipitated with A/G magnetic beads and an antibody overnight at 4 °C. After that, buffer was used to wash the samples, which were then allowed to cross-link. Finally, qPCR was used to verify the enrichment levels of the target proteins. The primer sequence information is listed in Table S3.

### Animal experiments

Male BALB/C nude mice and C57BL/6 mice were purchased from GemPharmatech Co., Ltd., and housed in a pathogen-free facility. For xenograft models, TFK1 cells (3 × 10^6^ per mouse) in 200 µL of PBS were injected subcutaneously into the left flank of each mouse. Each group included 8 mice. All of the mice were sacrificed after 4 weeks. In our research, all of the mice had tumors in the left flank. Tumor masses were measured with a digital Vernier caliper, and tumor volume (in cubic millimeters) was calculated using the following formula: width^2^ × length/2.

For C57BL/6 mice, we used a hydrodynamic method to rapidly and high-pressure inject 20 µg of the pT3EF1aH-myr-Akt plasmid (179909, Addgene), 20 µg of the pT3EF1aH-NICD1 plasmid (86500, Addgene) and 6 µg of the pCMV (CAT)T7-SB100 plasmid (34879, Addgene) into the tail vein of each mouse to construct a primary mouse CCA model [24]. In our research, we injected 20 µg of the pT3-EF1aH-MBD2 plasmid or the control plasmid pT3-EF1aH (vector) together with AKT/NICD1/SB100 to construct this model. We used the pT3-U6 plasmid as a vector to construct the pT3-U6-ABCB1-sh1 and pT3-U6-ABCB1-sh2 for knocking down the expression of ABCB1. The primers required for the constructs are presented in Table S3. Similarly, 20 µg of plasmid was mixed with the aforementioned plasmids and co-injected into the tail vein of the mice. Notably, all of the plasmid was suspended in 2 mL of saline, and the injection was finished in 5–7 s. Livers were collected after 4 weeks of hydrodynamic injection.

The method of drug dissolution was as follows: vehicle: 5% DMSO + 40% PEG 300 + 5% Tween 80 + 50% water; MM-102 was dissolved in vehicle; and cisplatin was dissolved in water. The mice were divided into 11 groups in 3 steps (5 mice per group): step 1: Vector + Vehicle, MBD2 + Vehicle, Vector + Cisplatin, and MBD2 + Cisplatin; step 2: MBD2 + Cisplatin, MBD2 + ABCB1-sh1 + Cisplatin, MBD2 + ABCB1-sh2 + Cisplatin; step 3: MBD2 + Vehicle, MBD2 + Cisplatin, MBD2 + MM-102 and MBD2 + Cisplatin + MM-102. All drugs were injected intraperitoneally into the mice 2 weeks after plasmid injection. Cisplatin was injected at a dose of 5 mg/kg on experimental Days 1, 5, 9, and 13. MM-102 was injected at a dose of 15 mg/kg for 7 days.

### Immunofluorescence (IF)

The cell samples were fixed with 4% formaldehyde for 15 min. PBS was used to wash the cells 3 times, and 0.5% Triton X-100 was added for 20 min at room temperature. After that, the samples were immersed in 5% BSA for 2 h and incubated with primary antibodies overnight at 4 °C. The samples were then incubated with secondary antibodies for 2 h at room temperature. Finally, DAPI was added for 3 min. The samples were observed under a laser confocal microscope.

### Statistical analysis

All of the experiments were performed independently 3 times. The data are expressed as the mean ± SD. IBM SPSS Statistics and Prism 8.0 were used for analysis. Differences between 2 groups were analyzed using the two-tailed Student’s t test, and differences among variance groups were analyzed by two-tailed ANOVA. The Kaplan–Meier method was used to generate survival curves. A p value less than 0.05 was considered statistically significant. * *P* < 0.05; ** *P* < 0.01; *** *P* < 0.001.

## Results

### MBD2 expression is upregulated in CCA

**Fig. 1:**
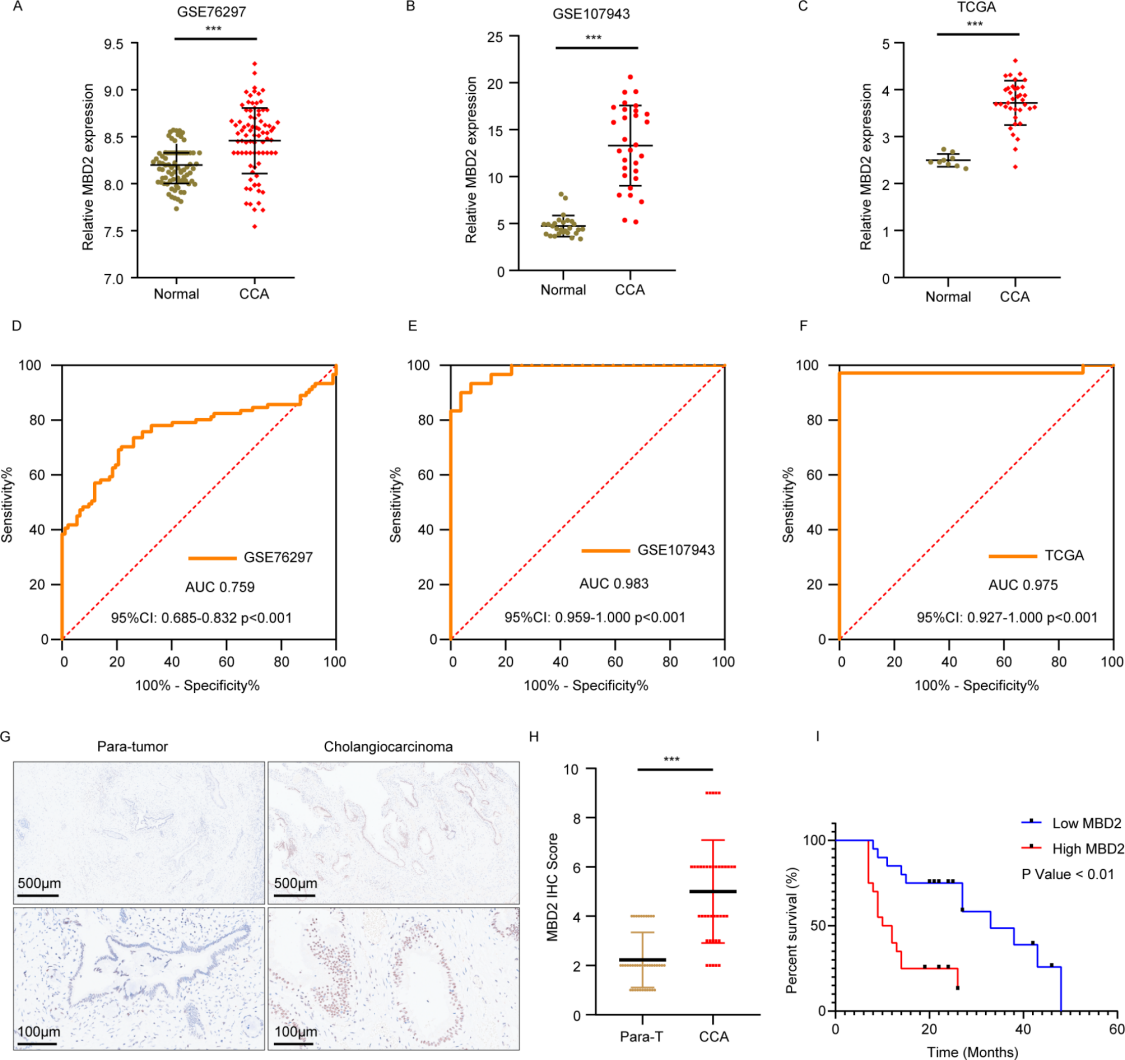
MBD2 expression is upregulated in CCA. **A**: Expression levels of MBD2 in GSE76297. **B**: Expression levels of MBD2 in GSE107943. **C**: Expression levels of MBD2 in TCGA. **D**: ROC curve of MBD2 from GSE76297. **E**: ROC curve of MBD2 from GSE107943. **F**: ROC curve of MBD2 from TCGA. **G**: Representative IHC images of MBD2 expression in 40 paratumor tissues and CCA tissues. **H**: IHC scores of MBD2 in paratumor tissues (*n* = 40) and CCA tissues (*n* = 40). **I**: Kaplan–Meier analysis of the overall survival rates in the low MBD2 expression group and the high MBD2 expression group. ***P < 0.001

To explore the expression of MBD2 in CCA, we used the public GEO (GSE76297, GSE107943) and TCGA databases. We found that MBD2 is significantly overexpressed in CCA tissues compared with normal bile duct tissues (Fig. 1A-C). In addition, receiver operating characteristic (ROC) curves of the GSE76297, GSE107943 and TCGA datasets were generated to evaluate the diagnostic value of MBD2 (Fig. 1D-F). The results indicated that the areas under the curve (AUCs) were 0.759, 0.983, and 0.975, respectively, meaning that MBD2 has a high predictive value for CCA. We further validated the results by immunohistochemical (IHC) staining in 40 pairs of CCA and paratumor tissues from our center, and the results were consistent with those from the TCGA and GEO databases (Fig. 1G-H). The clinicopathologic characteristics of these patients are shown in Table 1. These results indicated that the expression of MBD2 is correlated with the differentiation of CCA (*P* < 0.05). In addition, the overall survival of patients with CCA with relatively low MBD2 expression was longer than that of patients with high MBD2 expression (Fig. 1I). In summary, MBD2 is highly expressed in CCA, and patients whose MBD2 is upregulated have a worse prognosis.

**Table 1:** Clinicopathologic characteristics

Clinical characteristics	Numbers of patients	MBD2		*p*-Value
		Low expression	High expression	
Age (years)				0.74
≤ 60	14	6	8	
>60	26	14	12	
Gender				0.99
Male	29	15	14	
Female	11	5	6	
BMI				0.72
< 24	30	14	16	
≥ 24	10	6	4	
Differentiation				0.02*
I^#^	24	16	8	
II^#^	16	4	12	
Stage				0.30
I and II	28	12	16	
III and IV	12	8	4	

Low expression: IHC staining index < 6; High expression: IHC staining index ≥ 6

I^#^: Well differentiation + Well to moderately differentiation + Moderately differentiation

II^#^: Moderately to low differentiation + Low differentiation

### MBD2 promotes the proliferation, migration and chemoresistance of CCA

**Fig. 2:**
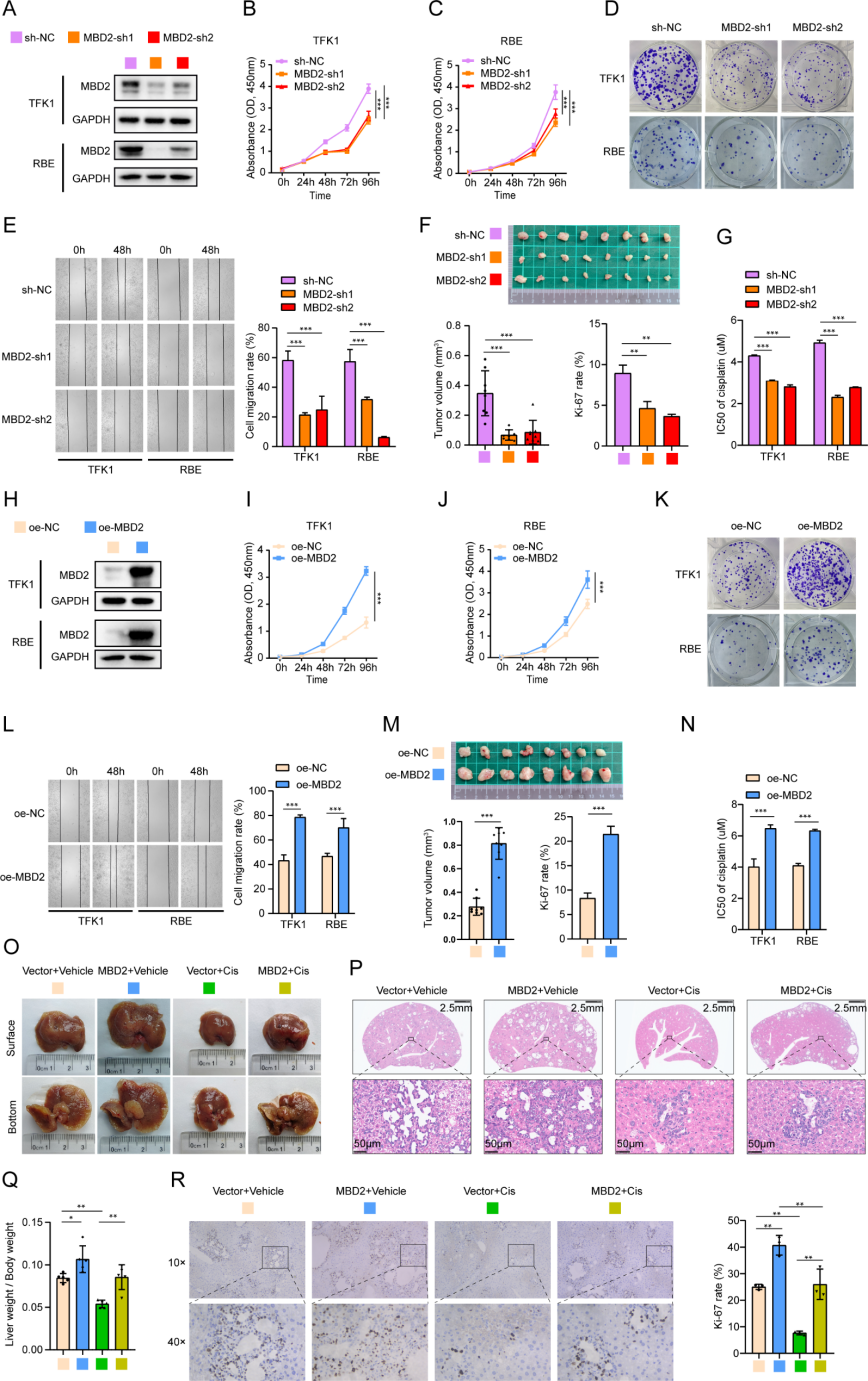
MBD2 promotes the proliferation, migration and chemoresistance of CCA. **A**: Knockdown of MBD2 in TFK1 cells and RBE cells was validated by western blotting. **B**-**D**: The proliferation ability of MBD2-knockdown TFK1 and RBE cells was detected by a CCK-8 assay (B and C) and a plate cloning assay (D). **E**: Representative images of the wound healing assay used to detect the migration of MBD2-knockdown TFK1 and RBE cells and the statistical volume. **F**: Tumors of xenograft mice models derived from MBD2-knockdown TFK1 cells. The image below shows the statistical analysis of the tumor volume and the Ki-67 rate. **G**: IC50 values of MBD2 knockdown in CCA cells treated with cisplatin. **H**: Overexpression of MBD2 in TFK1 and RBE cells was validated by western blotting. I-K: The proliferation abilities of MBD2-overexpressing TFK1 and RBE cells were detected by a CCK-8 assay (I and J) and a plate cloning assay (**K**). **L**: Representative images of a wound healing assay in which MBD2 was overexpressed in TFK1 and RBE cells and the statistical analysis of the results. **M**: The tumor of xenograft mice models derived from MBD2-overexpressing TFK1 cells. The image below shows the statistical analysis of the tumor volume and the Ki-67 rate. **N**: IC50 values of MBD2 overexpression in TFK1 and RBE cells exposed to cisplatin. **O**: Representative images of livers in primary CCA mice models. Cis: cisplatin. **P**: HE staining of the liver in primary CCA mice models. **Q**: The ratio of liver weight to body weight in primary CCA mice models. **R**: IHC staining of Ki-67 in liver of primary CCA mice models. The right panel shows the statistical of Ki-67 rate. *P<0.05, **P<0.01, ***P<0.001

Because MBD2 is upregulated in CCA, we investigated the impact of MBD2 in CCA cells. Lentivirus-mediated transfection of MBD2-shRNAs (MBD2-sh1, MBD2-sh2) and MBD2 (oe-MBD2) was used to induce the downregulation and overexpression of MBD2 in TFK1 and RBE (Fig. 2A and H). The results of the Cell Counting Kit-8 (CCK-8), plate cloning and wound healing assays revealed that MBD2 knockdown significantly suppressed the proliferation and migration of CCA cells (Fig. 2B-E, Figure S1K-L), whereas the overexpression of MBD2 promoted these effects (Fig. 2I-L, Figure S1M-N). Next, in a subcutaneous xenograft model, MBD2 knockdown significantly inhibited the proliferation of tumors and decreased the expression of Ki-67 (Fig. 2F and Figure S2A). The opposite effect was found with the overexpression of MBD2 (Fig. 2M and Figure S2B).

Cisplatin is a platinum-containing drug that is widely used in cancer therapy. The main mechanism of the antitumor effect of cisplatin is that it can interact with DNA and disrupt its structure [25]. Notably, cisplatin plus gemcitabine is the basic treatment for patients with CCA [26]. Therefore, we used a CCK-8 assay to assess the effect of MBD2 on the cisplatin sensitivity of CCA cells. The results indicated that knocking down MBD2 increased the sensitivity of TFK1 and RBE to cisplatin, resulting in lower IC50 values (Fig. 2G, Figure S1A-B), whereas the opposite outcomes were observed in CCA cells overexpressing MBD2 (Fig. 2N, Figure S1C-D). In addition, we established primary CCA mice models by hydrodynamic transfection of Akt and NICD1 plasmids into C57BL/6 mice via the tail vein. MBD2-expressing or vector plasmid was coinjected with the Akt/NICD1 plasmid (10 mice/group) to observe the impact of MBD2 on CCA in vivo. Two weeks after injection, cisplatin (5 mg/kg) was intraperitoneally injected into 5 mice in the MBD2 group and 5 mice in the vector group to evaluate the impact of MBD2 on cisplatin resistance, and vehicle was injected into the other mice. All of the mice were euthanized after 4 weeks of plasmid injection. Based on the liver specimen and hematoxylin and eosin (HE) staining results, MBD2 + Vehicle mice presented a greater tumor load than Vector + Vehicle mice did. After treatment with cisplatin, a few tumors were still found in the livers of the MBD2 + Cis group, whereas almost no tumors were found in the livers of the Vector + Cis group (Fig. 2O-P). Owing to the heavy tumor burden, the MBD2 + Vehicle group had a greater liver-to-body weight ratio than did the Vector + Vehicle group. This ratio in the MBD2 + Cis group was also greater than that in the Vector + Cis group (Fig. 2Q). IHC staining revealed that the MBD2 + Vehicle group had the highest Ki-67 positivity rate, and this rate was greater in the MBD2 + Cis group than in the Vector + Cis group (Fig. 2R). Overall, these results indicate that MBD2 not only accelerates the progression of CCA but also induces resistance to cisplatin.

### MBD2 regulates the expression of ABCB1 not through NURD

**Fig. 3:**
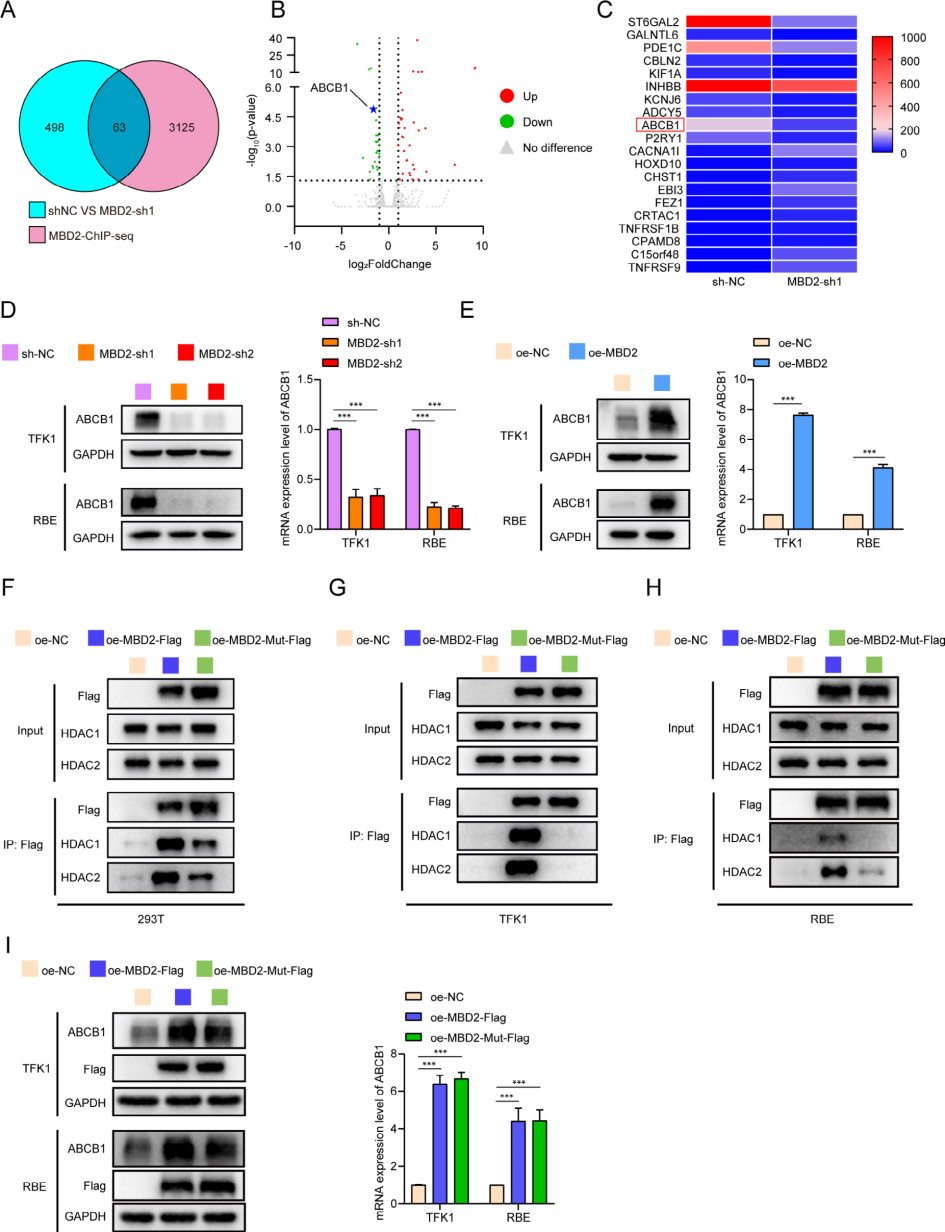
MBD2 regulates the expression of ABCB1 not through NURD. **A**: Venn diagram of the RNA-seq (GSE275477, negative control with MBD2 knockdown in TFK1 cells) with the ChIP-seq data of MBD2 from the GEO database (GSE41006). **B**: Volcano map of the RNA-seq data (negative control with MBD2 knockdown in TFK1 cells) with the ChIP-seq data of MBD2 from GEO database (GSE41006). **C**: Heatmap of the top 10 genes among up-regulated and down-regulated genes from the intersection of RNA-seq and ChIP-seq data. **D**-**E**: The protein and mRNA levels of ABCB1 was examined by western blot and RT-PCR in MBD2-knockdown and MBD2-overexpressing cells. **F**-**H**: IP was performed to detect the interaction of MBD2 or MBD2-Mut with HDAC1/2 in 293T, TFK1 and RBE cells. **I**: ABCB1 expression was examined by western blot and RT-PCR in oe-MBD2 and oe-MBD2-Mut cells. ***P < 0.001

To explore the molecular characteristics of MBD2 in CCA, RNA-seq analysis of MBD2-knockdown and negative control TFK1 cells was performed (GSE275477). In addition, we compared the RNA-seq results (*P* < 0.05, |FD|>1) with the ChIP-seq results (obtaining from Cistrome Data Browser) for MBD2 (putative score ≥ 1) from the GEO database (GSE41006) (Fig. 3A-B). We then sorted the top ten genes that were upregulated or downregulated based on the log2FC values, and the results are shown in Fig. 3C. Despite having high log2FC values, the top 10 genes in the upregulated group were expressed at extremely low levels in CCA cells according to RNA-seq; therefore, we focused mainly on the downregulated group. We found that the chemoresistance-related gene ABCB1 was significantly inhibited after MBD2 was knocked down (log2FC=-1.63, *P* < 0.05, ranking 9th among the downregulated genes) (Fig. 3C). As a drug transporter, ABCB1 can excrete a wide range of drugs out of the cell, playing an important role in the process of chemoresistance. Western blotting and RT-PCR were performed to validate the above results, and we found that knocking down MBD2 inhibited the expression of ABCB1, whereas the opposite effect was observed in CCA cells overexpressing MBD2 (Fig. 3D-E). These results indicate that MBD2 may promote the expression of ABCB1.

However, MBD2 always induces transcriptional silencing by directly binding to methylated CpG dinucleotides, inducing chromatin remodeling or recruiting transcriptional complexes such as NURD [27]. NURD is a multisubunit protein complex that includes the histone deacetylase HDAC1/2, the ATP-dependent remodeling enzymes CHD3/4, the histone chaperones RbAp46/48, GATAD2a (p66α), GAGAD2b (p66β), the specific DNA-binding proteins MTA1/2/3 and the CpG-binding proteins MBD2/3 [28]. Among numerous subunits, HDAC1/2 are responsible for histone deacetylation and are the key enzymes in the NURD complex that induce transcriptional inhibition. Although deacetylation is largely associated with gene repression, genome-wide ChIP-seq has shown that HDAC1/2 are also enriched at transcriptionally active loci [29]. Therefore, it was necessary to clarify whether the regulation of ABCB1 by MBD2 depends on NURD. We constructed a mutation vector with Flag tags for MBD2 (R286E, L287A), named oe-MBD2-Mut. The double amino acid mutation of MBD2 results in a disrupted interaction with NURD [27]. Then, we transferred oe-MBD2-Mut with Flag tags into 293T, TFK1 and RBE cells. Immunoprecipitation (IP) was performed to detect the interactions with HDAC1 and HDAC2. Compared with that of oe-MBD2, the interaction of MBD2 with HDAC1/2 was significantly disrupted in the oe-MBD2-Mut group (Fig. 3F-H). Next, we tested the effect of the MBD2 mutation on the expression of ABCB1 by western blotting and RT-PCR. However, mutation of MBD2 did not affect the expression of ABCB1 compared with that in the oe-MBD2 group (Fig. 3I), which indicated that disrupting the interaction of MBD2 and NURD does not affect the regulation of ABCB1 by MBD2. In addition, the results of western blotting and RT-PCR showed that MBD2 has no effect on the expression of HDAC1/2 (Figure S3A-C).

To investigate whether MBD2 enhances the chemoresistance of CCA cells by regulating ABCB1, we overexpressed ABCB1 in MBD2 knockdown CCA cells (Figure S4A-B). The results suggested that ABCB1 could reverse the increased sensitivity to cisplatin induced by MBD2 knockdown (Figure S4C-D). Then we knocked down the expression of ABCB1 in the AKT/NICD1/MBD2 model, and the MBD2 + ABCB1-sh1/2 group exhibited enhanced cisplatin therapeutic efficacy compared to MBD2 group, with fewer liver lesions, a lower liver-to-body weight ratio, and Ki67 rate (Figure S4E-H). These results indicate that MBD2 does not regulate ABCB1 through NURD.

### MBD2 directly interacts with WDR5 in CCA cells

**Fig. 4:**
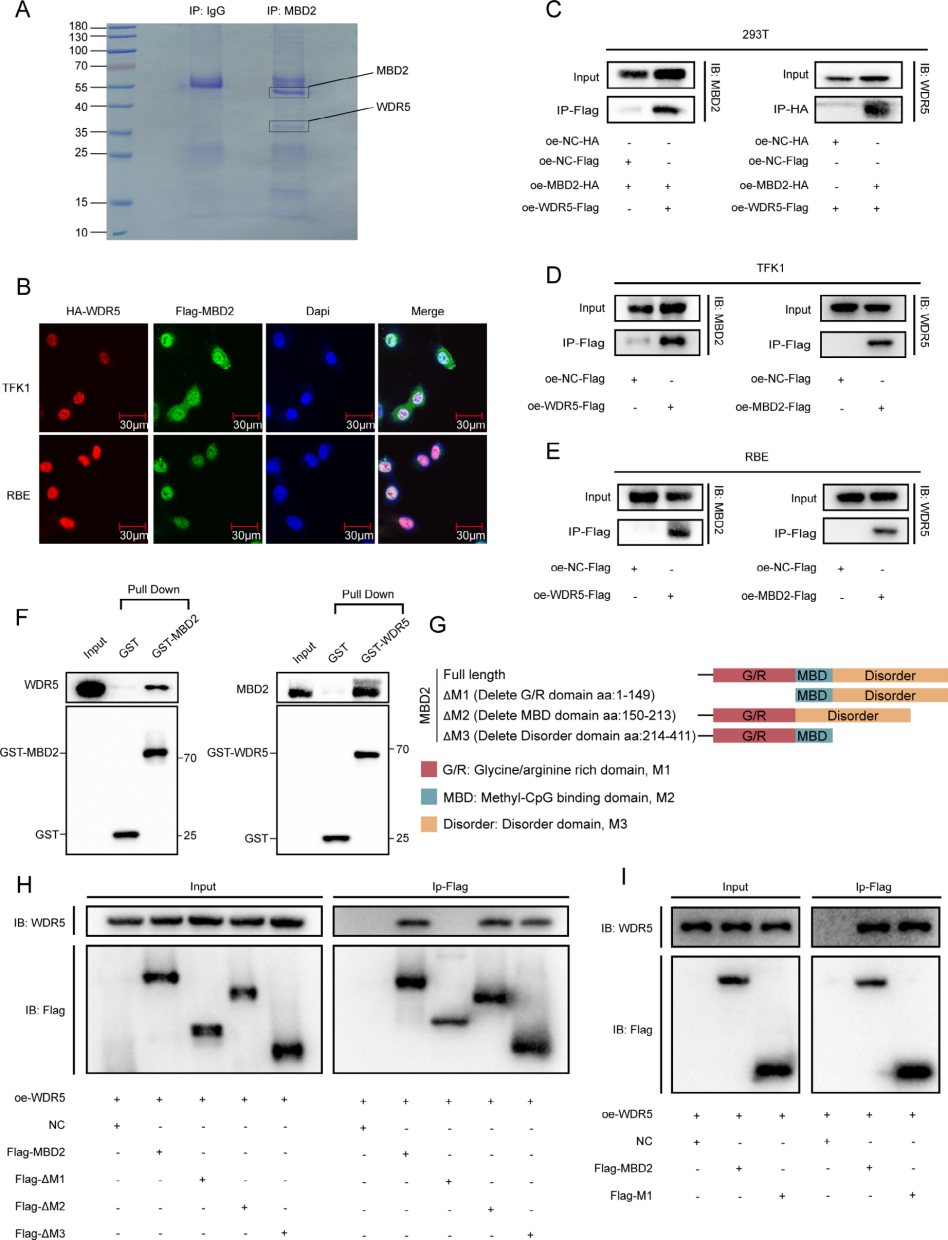
MBD2 directly interacts with WDR5 in CCA cells. **A**: The gel of the negative control group and MBD2-IP group from 293T cells. The protein samples were separated by SDS-PAGE and stained with Coomassie brilliant blue. The protein masses are shown in the left. **B**: Co-localization of MBD2 and WDR5 was detected by immunofluorescence. **C**-**E**: The interaction of MBD2 with WDR5 was detected by IP in 293T, TFK1 and RBE cells. **F**: The direct interaction of MBD2 with WDR5 was confirmed by GST pull-down. **G**: The schematic diagram of the domain organization of MBD2 and its truncated mutants. **H**: 293T cells was transfected with the full length or the truncated mutants of MBD2. Co-IP was used to detect which of the truncated fragment of MBD2 bound to WDR5. **I**: IP was used to detect the binding ability between Flag-M1 fragments and WDR5

To explore the specific mechanism of MBD2-mediated upregulation of ABCB1, we performed coimmunoprecipitation mass spectrometry (co-IP-MS) experiments in 293T cells (Fig. 4A and Table S2). A total of 795 proteins that interact with MBD2 were discovered. By analyzing these proteins, we discovered that WDR5, an essential protein that assists KMT2A in catalyzing H3K4 trimethylation, is a marker for transcriptional promotion. Previous studies have shown that WDR5 plays an important role in promoting tumor progression and chemoresistance [21, 30]. Therefore, we speculated that MBD2 regulates ABCB1 by forming a complex with WDR5. IF confirmed the colocalization of MBD2 and WDR5 in the nucleus (Fig. 4B). Coimmunoprecipitation of 293T, TFK1 and RBE cells confirmed that MBD2 can interact with WDR5 (Fig. 4C-E). In addition, a GST pull-down assay revealed that MBD2 can directly interact with WDR5 (Fig. 4F). Finally, we studied the detailed region of the MBD2 combination of WDR5. We divided MBD2 into three regions: the GR-rich region (M1, amino acids 1–149), the MBD region (M2, amino acids 150–213), and the disordered structure (M3, amino acids 214–411). We first deleted these 3 regions respectively on the full-length MBD2 and obtained segments of ΔM1, ΔM2, and ΔM3 (Fig. 4G). We subsequently conducted co-IP to verify its ability to bind with WDR5. The results indicated that the interaction with WDR5 was disrupted in the ΔM1 segment (Fig. 4H). We then induced the vector containing the M1 segment into 293T cells, and the results of co-IP revealed that M1 was responsible for the interaction between MBD2 and WDR5 (Fig. 4I). These results indicated that MBD2 can directly interact with WDR5 through GR-rich regions.

### WDR5 promotes the proliferation, migration and chemoresistance of CCA

**Fig. 5:**
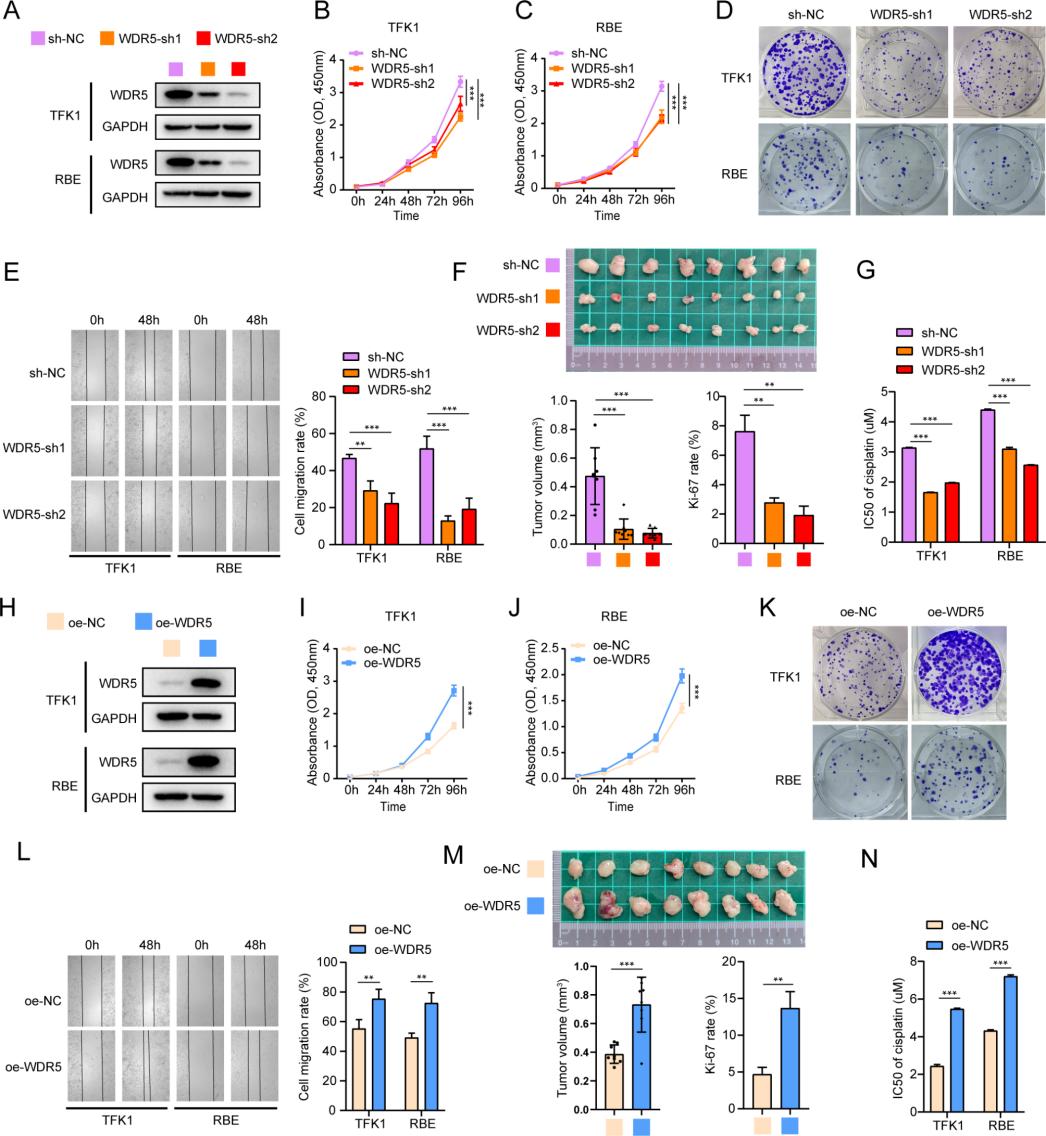
WDR5 promotes the proliferation, migration and chemoresistance of CCA. **A**: Western blotting was performed to detect the effect of WDR5 knockdown in TFK1 and RBE cells. **B**-**D**: The proliferation abilities of TFK1 and RBE cells with WDR5 knockdown was detected by CCK-8 assay (**B** and **C**) and plate cloning assay (**D**). **E**: Representative images of wound healing assay used to detect the migration of TFK1 and RBE cells in which WDR5 was knocked down and the statistical volume. **F**: Tumors of xenograft mice models derived from TFK1 cells with WDR5 knockdown. The image below shows the statistical analysis of the tumor volume and the Ki-67 rate. **G**: IC50 values of CCA cells in response to cisplatin upon WDR5 knockdown. **H**: Overexpression of WDR5 in TFK1 and RBE cells was validated by western blotting. I-K: The proliferation abilities of TFK1 and RBE cells overexpressing WDR5 were detected by CCK-8 assay (**I** and **J**) and plate cloning assay (**K**). **L**: Representative images of wound healing assay in which WDR5 was overexpressed in TFK1 and RBE cells and the statistical volume. **M**: The tumor of xenograft mice models derived from TFK1 cells with WDR5 overexpression. The image below is the statistical of tumor volume and Ki-67 rate. **N**: IC50 values of TFK1 and RBE cells response to cisplatin upon WDR5 overexpression. **P < 0.01, ***P < 0.001

Owing to the interaction between WDR5 and MBD2 and the important role of WDR5 in various tumors, we began to study the impact of WDR5 on CCA cells. CCK-8, plate cloning and wound healing assays revealed that knockdown of WDR5 (Fig. 5A) inhibited the proliferation and migration abilities of CCA cells (Fig. 5B-E, Figure S1O-P), whereas overexpression of WDR5 (Fig. 5H) promoted these effects (Fig. 5I-L, Figure S1Q-R). In addition, in the subcutaneous xenograft model, WDR5 knockdown inhibited tumor growth and decreased the Ki-67 expression rate compared with that in the control group (Fig. 5F and Figure S2C), whereas WDR5 overexpression had the opposite effects (Fig. 5M and Figure S2D). Finally, the results of the CCK-8 assay also indicated that downregulation of WDR5 decreased the IC50 of cisplatin in CCA cells (Fig. 5G and Figure S1E-F). However, the results of overexpressing WDR5 were exactly opposite to all the above results (Fig. 5N and Figure S1G-H). Taken together, these data demonstrated that WDR5 functions as an oncogene to facilitate the proliferation, migration and chemoresistance of CCA cells.

### MBD2 activates the expression of ABCB1 through the WDR5-KMT2A complex

**Fig. 6:**
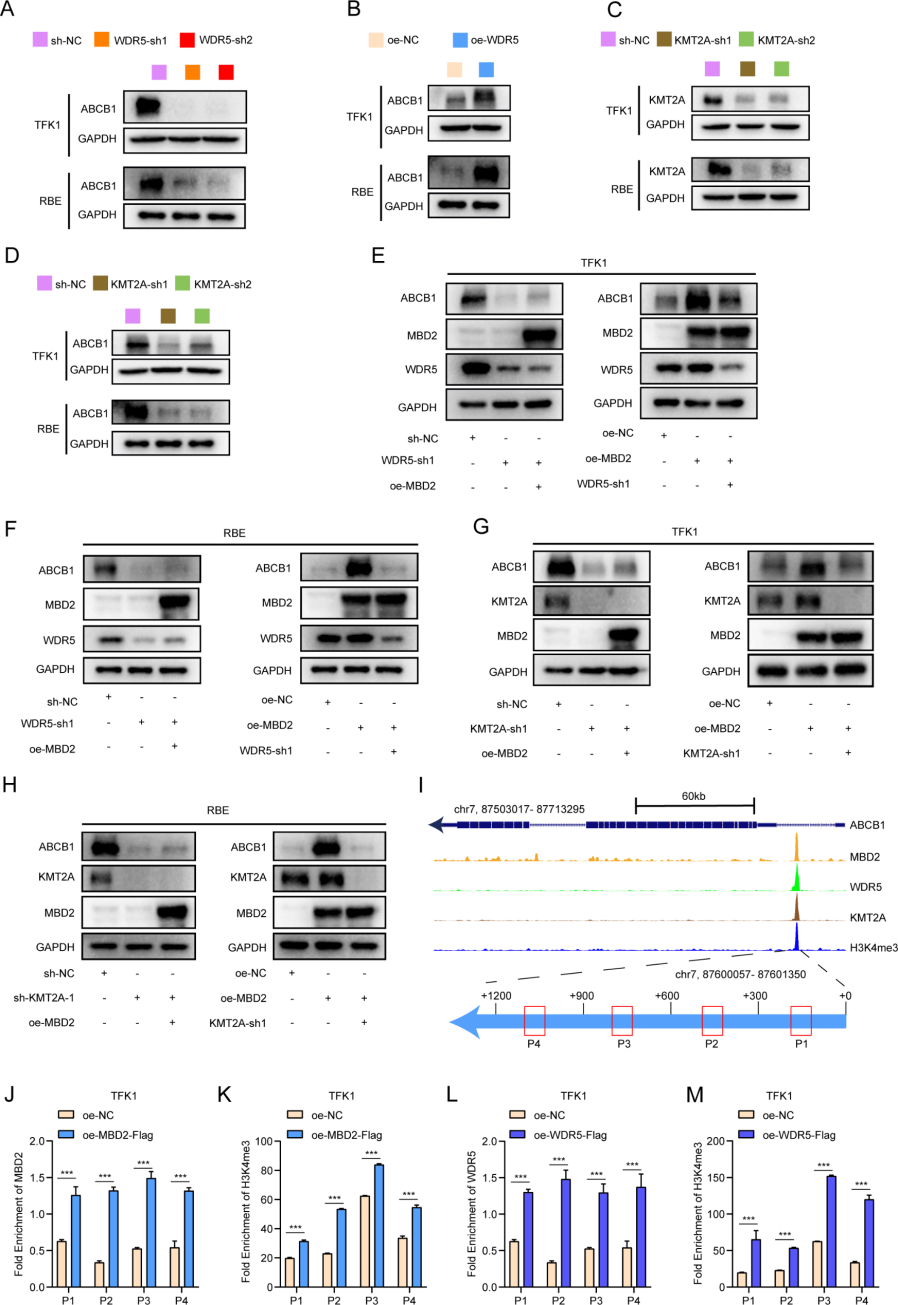
MBD2 activates the expression of ABCB1 through the WDR5-KMT2A complex. **A**-**B**: The protein levels of ABCB1 was examined by western blot in WDR5 knockdown and overexpression cells. **C**: Western blotting was used to detect the effect of KMT2A knockdown in TFK1 and RBE cells. **D**: The protein levels of ABCB1 was examined by western blot in KMT2A knockdown cells. **E**-**F**: The protein levels of ABCB1 was detected by western blot upon overexpression of MBD2 and knockdown of WDR5. **G**-**H**: The protein levels of ABCB1 was detected by western blot upon overexpression of MBD2 and knockdown of KMT2A. **I**: The GEO database indicates binding peaks of MBD2, WDR5, and KMT2A on the ABCB1 gene. The below panel is the schematic diagram of binding peak of ABCB1 and the ChIP-qPCR product (P1, P2, P3, P4). **J**-**M**: The ChIP-qPCR was used to examine the binding of MBD2, WDR5 and H3K4me3 to ABCB1 in MBD2 or WDR5 overexpression TFK1 cells. Anti-Flag antibody was used for ChIP-qPCR to detect the enrichment of MBD2-Flag or WDR5-Flag in the binding region; Anti-H3K4me3 antibody was used for ChIP-qPCR to detect the enrichment of H3K4me3 in the binding region. ***P < 0.001

Given the results above, we determined the role of WDR5 in mediating the expression of ABCB1. The results suggested that WDR5 knockdown inhibited the expression of ABCB1 in TFK1 and RBE cells, whereas overexpression of WDR5 had the opposite effect (Fig. 6A-B). Previous studies have shown that WDR5 is an essential protein that combines with KMT2A to catalyze H3K4 trimethylation [15–17]. The impact of KMT2A in CCA cells was detected by western blotting, and the results revealed that knockdown of KMT2A significantly decreased the expression of ABCB1 (Fig. 6C-D).

To confirm that MBD2 regulates ABCB1 through WDR5 and KMT2A, a rescue experiment was performed by western blotting. The knockdown of WDR5 disrupted the activation of ABCB1 through the overexpression of MBD2 in CCA cells (Fig. 6E-F). Similarly, knockdown of KMT2A in CCA cells also blocked the effect of MBD2 on ABCB1 (Fig. 6G-H). These results indicate the necessity of WDR5 and KMT2A in the regulation of ABCB1 expression by MBD2.

To demonstrate that the MBD2-WDR5-KMT2A complex coregulates the expression of ABCB1 through H3K4 trimethylation, we analyzed GEO datasets (GSE41006, GSE60897, GSE83671 and GSE101646). The data indicated that MBD2, WDR5, KMT2A and H3K4me3 share a common binding peak (Ch7, 87600057–87601350) in the ABCB1 promoter (Fig. 6I). Then we divided the peak into four segments (P1-P4), each approximately 300 bp, and performed ChIP-qPCR to validate the above results in TFK1 and RBE cells. After MBD2 and WDR5 were overexpressed, the enrichment of these proteins in the ABCB1 promoter significantly increased compared with that in the negative control, as did H3K4me3 (Fig. 6J-M, Figure S5A-D).

Finally, we detected the mRNA expression level of WDR5 and KMT2A in 40 pairs of CCA and paratumor tissues from our center. The results indicated that WDR5 and KMT2A were overexpressed in CCA tissues compared with paratumor tissues (Figure S6A-B). In addition, the overall survival of patients with CCA with relatively low WDR5 or KMT2A expression was longer than that of patients with high expression (Figure S6C-D). These results demonstrate that MBD2 promotes the expression of ABCB1 by recruiting WDR5-KMT2A to the promoter of ABCB1, where it catalyzes H3K4 trimethylation.

### Targeted inhibition of the WDR5-KMT2A interaction by MM-102 weakens the proliferation, migration, and chemoresistance of CCA

**Fig. 7:**
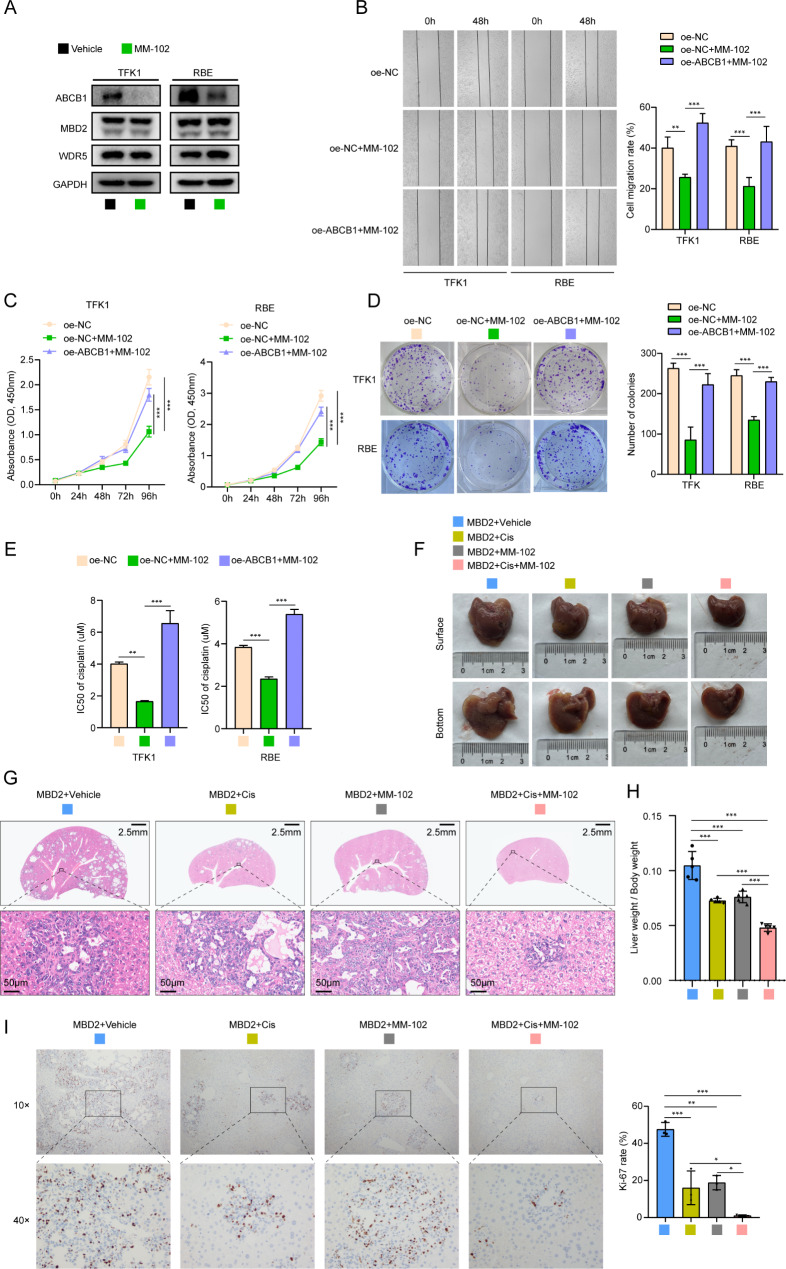
Targeted inhibition of the WDR5-KMT2A interaction by MM-102 weakens the proliferation, migration, and chemoresistance of CCA. **A**: The protein levels of ABCB1, MBD2 and WDR5 was examined by western blot in TFK and RBE cells supplemented with MM-102. **B**: The representative image of wound healing assay to detect the migration of TFK1 and RBE cells added with MM-102. The right panel is the statistical volume. C-D: The proliferation abilities of TFK1 and RBE cells supplemented with MM-102 was detected by CCK-8 assay (**C**) and plate cloning assay (**D**). **E**: IC50 value of TFK1 and RBE cells to cisplatin upon added with MM-102. **F**: Representative image of liver in primary CCA mice models treated with cisplatin or MM-102. Cis: cisplatin. **G**: HE staining of the liver in primary CCA mice models treated with cisplatin or MM-102. Cis: cisplatin. **H**: The ratio of liver weight to body weight in primary CCA mice models. **I**: IHC staining of Ki-67 in liver of primary CCA mice models. The right panel is the statistical of Ki-67 rate. *P < 0.05, **P < 0.01, ***P < 0.001

Owing to the regulation of ABCB1 by MBD2 through the WDR5-KMT2A complex and because KMT2A is responsible for H3K4 trimethylation, we attempted to search for targeted inhibitors to provide new ideas for clinical treatment. MM-102, a mimetic peptide of KMT2A, has high affinity for WDR5 and can effectively inhibit the interaction between WDR5 and KMT2A [31]. We first applied MM-102 to CCA cells and found that the expression of ABCB1 was significantly inhibited, with no effect on the expression of MBD2 and WDR5 (Fig. 7A). The results of the CCK-8, plate colony formation and wound healing assays also indicated that MM-102 could significantly suppress the proliferation, migration and chemoresistance of CCA cells, whereas overexpressing ABCB1 reversed all these effects (Fig. 7B-E, Figure S1 I-J). We subsequently used primary CCA mice models (Akt/NICD1/MBD2 mice) to test the therapeutic effect of MM-102 in vivo. These mice were treated with vehicle, cisplatin monotherapy, MM-102 monotherapy or cisplatin combined with MM-102. Four weeks after plasmid injection, the mice were euthanized, and it was found that both cisplatin and MM-102 had the ability to inhibit tumor progression. However, combination of the cisplatin and MM-102 had the best therapeutic effect, with liver tumors almost disappearing (Fig. 7F-G). Compared with the ratios in the other groups, the liver weight-to-body weight ratio in the MBD2 + Cis + MM-102 group was lower (Fig. 7H). IHC staining also revealed a significant decrease in the Ki-67 rate in this group (Fig. 7I). These results indicate that MM-102 may effectively increase the sensitivity of CCA to cisplatin in vivo.

**Fig. 8:**
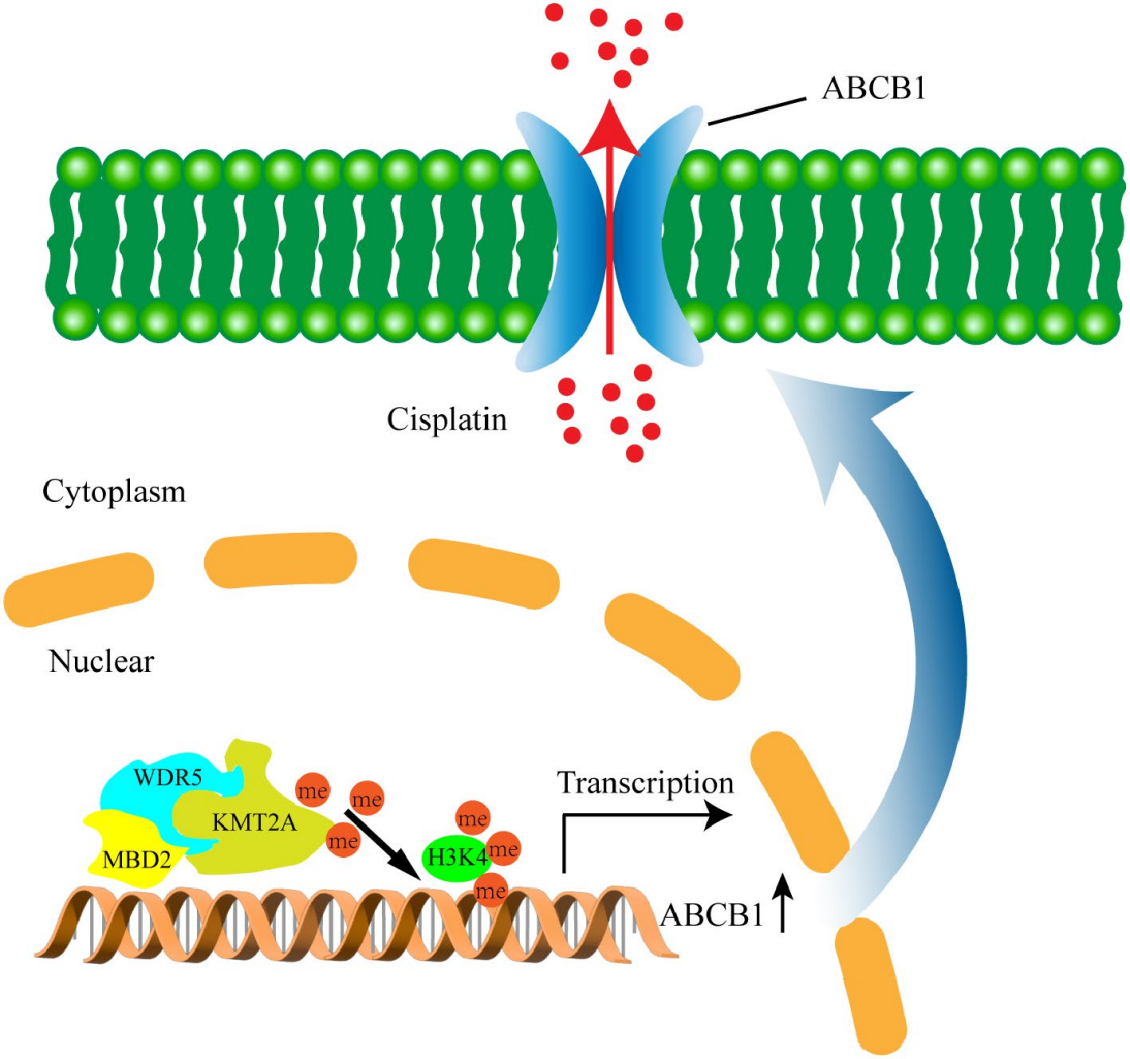
Schematic of a model for the major molecular mechanisms. MBD2 forms a complex with WDR5-KMT2A through their interaction, promoting the H3K4 trimethylation of specific regions of the ABCB1 promoter, which enhances ABCB1 expression and contributes to the chemoresistance, proliferation, and migration capabilities of cholangiocarcinoma

Taken together, the results of this study elucidate the potential mechanism underlying the overexpression of MBD2 in CCA and its contributions to tumor progression and chemoresistance. MBD2 activates the expression of ABCB1 through recruiting the WDR5-KMT2A complex to its promoter, catalyzing the trimethylation of H3K4. An inhibitor of WDR5-KMT2A (MM-102) can increase the sensitivity of CCA to cisplatin, which may provide ideas for the treatment of chemoresistance in CCA (Fig. 8).

## Discussion

MBD proteins are key epigenetic regulatory factors that affect the transcription of genes through a variety of mechanisms. MBD2, a classic protein, can bind to DNA and recruit NURD to induce transcriptional silencing [32]. However, MBD2 can also bind to promoters and activate chromatin marks, subsequently promoting transcription [9, 33]. MBD2 plays important roles in various tumors [34–37], especially acute myeloid leukemia [12]; however, few reports on its role in the CCA exist. To clarify the roles of MBD2 in CCA, we first analyzed the expression of MBD2 in CCA and normal tissues in the public GEO (GSE76297, GSE107943) and TCGA databases. The results revealed that MBD2 was highly expressed in CCA tissues. The ROC curves of the GSE76297, GSE107943 and TCGA datasets indicated that MBD2 has good diagnostic efficacy for CCA. IHC staining of 40 pairs of tissues from our center also validated these results. In addition, we followed these 40 patients with CCA and reported that patients with low MBD2 expression had a relatively high overall survival rate. These data suggest that MBD2 may play an important role in CCA.

To further investigate the role of MBD2 in CCA, we performed lentiviral-mediated transfection to change the expression of MBD2 in CCA cells. A series of loss- and gain-of-function experiments revealed that MBD2 knockdown inhibited and that MBD2 overexpression promoted proliferation, migration and chemoresistance in CCA cells. Subcutaneous xenograft models also produced the same results. In addition, we used hydrodynamic transfection to establish primary CCA mice models and then treated them with cisplatin to assess the function of MBD2 in chemoresistance. These results indicate that MBD2 promotes the chemoresistance of CCA. Therefore, MBD2, as an oncogene, promotes the progression of CCA.

Through RNA-seq combined with ChIP-seq data from the GEO database, we identified the top ten genes for upregulation or downregulation. Owing to the extremely low expression of the top 10 genes in the upregulated group according to RNA-seq, we focused mainly on the downregulated group. Chemoresistance is an important factor leading to poor treatment for CCA. Because of the important role of ABCB1 in chemoresistance, we first examined the impact of MBD2 on ABCB1. Western blot and RT-PCR analysis revealed that MBD2 could activate the expression of ABCB1. However, early studies of MBD2 focused on its functions in transcriptional repression. The first model of MBD2 proposed that MBD2 recruits NURD to methylated regions of genes and induces transcriptional silencing through histone deacetylation [32]. This regulatory mechanism, which involves the transcriptional inhibition of multiple target genes, has been reported in numerous studies [38–40]. Therefore, it was necessary to clarify whether the promotion of ABCB1 transcription by MBD2 is related to NURD. We blocked the binding of MBD2 to NURD by mutating the amino acid site (R286E, L287A) of MBD2 according to Desai MA [27]. The results of co-IP in 293T, TFK1 and RBE cells confirmed that Mut-MBD2 significantly lost its ability to bind with HDAC1/2. Moreover, Mut-MBD2 could still promote the expression of ABCB1, meaning that MBD2 does not activate the transcription of ABCB1 through NURD. Our subsequent research revealed that the overexpression of ABCB1 can notably reverse the increased sensitivity to cisplatin induced by the knockdown of MBD2 in CCA cells. Similarly, reducing the expression of ABCB1 in AKT/NICD1/MBD2 CCA mice model enhanced the therapeutic efficacy of cisplatin.

To better understand the mechanism of MBD2 regulating ABCB1, we performed co-IP-MS of MBD2 and identified WDR5 from the results. Emerging evidence has shown that WDR5 consists of seven WD40 repeat domains, which interact with the N-terminal end of the histone H3 tail [41]. Moreover, the binding pocket of WDR5 could interact with the H3K4 methyltransferase enzyme KMT2A. As the core subunit of MLL histone H3K4 methyltransferase complexes, WDR5 is necessary for the methylation of H3K4. Disruption of WDR5 results in blockage of H3K4 methylation [16, 42]. Histone H3K4 methylation is common in the regulation of gene expression and is always associated with transcriptional activation [43]. Through IF, co-IP and GST pull-down assays, we found that MBD2 could directly interact with WDR5 and that the GR-rich region of MBD2 was responsible for this interaction.

Previous studies have shown that WDR5 serves as an oncogene in a variety of cancers [18, 20, 44, 45]. In our study, we found that WDR5 also promoted the proliferation, migration and chemoresistance of CCA cells. WDR5 has also been shown to interact with KMT2A and activate the transcription of target genes through the trimethylation of H3K4 [15–17]. Our results revealed that WDR5 could also activate the expression of ABCB1. Knocking down WDR5 could block the transcriptional activity of ABCB1 via MBD2. Moreover, it could also reduce the expression level of ABCB1, which is already activated by the overexpression of MBD2. Similarly, knocking down the expression of KMT2A effectively blocked the transcriptional activation of ABCB1 by MBD2, similar to the results above. The public ChIP-seq database revealed that MBD2, WDR5, KMT2A and H3K4me3 share a common binding peak in the promoter of ABCB1. Importantly, we checked the other 9 genes in the downregulated group and found that no genes presented a common binding peak for MBD2, WDR5 or KMT2A. We then designed four pairs of primers targeting this binding peak for ChIP‒qPCR. These results confirmed that MBD2 and WDR5 can bind to this region of ABCB1 and increase the enrichment of H3K4me3 to promote transcription. Our results indicated that MBD2 activated the transcription of ABCB1 through the WDR5-KMT2A complex.

MM-102 has been reported to effectively inhibit the interaction between WDR5 and KMT2A [31]. Here, we found that MM-102 could suppress the proliferation, migration and chemoresistance in CCA cells. In addition, treating MM-102 cells with CCA significantly decreased the expression of ABCB1. Moreover, the overexpression of ABCB1 can reverse the inhibitory effect induced by MM-102 in CCA cells. Although primary CCA mice models with MBD2 overexpression exhibited cisplatin resistance, the combination of cisplatin and MM-102 could effectively eliminate tumors in the liver and decrease the Ki-67 rate compared with treatment with cisplatin or MM-102 alone. These findings indicate that MM-102 can increase the sensitivity of CCA to cisplatin.

## Conclusions

In summary, our study demonstrated that the MBD2/WDR5/KMT2A-ABCB1 axis plays crucial roles in CCA progression and chemoresistance. Targeting this axis may decrease the chemoresistance of CCA and suggest a new strategy for CCA treatment.

## Electronic supplementary material

Below is the link to the electronic supplementary material.

Supplementary Material 1: Table S1. The primary antibodies used in this study.

Supplementary Material 2: Table S2 The results of co-IP-MS

Supplementary Material 3: Table S3 The sequence of primers

Supplementary Material 4: Figure S1 The viability of CCA cells to cisplatin and the statistical chart of plate clones. A-D: TFK1 and RBE cells knocking down or overexpressing MBD2 were treated with cisplatin, CCK-8 was used to detect the cells viability. E-H: TFK1 and RBE cells knocking down or overexpressing WDR5 were treated with cisplatin, CCK-8 was used to detect the cells viability. I-J: TFK1 and RBE cells were treated with MM-102 and cisplatin, CCK-8 was used to detect the cells viability. K-N: The statistical volume of plate cloning assay in MBD2 knockdown or overexpression cells. O-R: The statistical volume of plate cloning assay in WDR5 knockdown or overexpression cells. **P<0.01, ***P<0.001.

Supplementary Material 5: Figure S2 The IHC of tumor from xenograft mice models. A-B: The IHC staining of Ki-67 in MBD2 knockdown and overexpression xenograft tumor derived from TFK1 cells. C-D: The IHC staining of Ki-67 in WDR5 knockdown and overexpression xenograft tumor derived from TFK1cells.

Supplementary Material 6: Figure S3 MBD2 does not affect the expression of HDAC1/2A-C: Western blot (A) and RT-PCR (B and C) were used to detect the effect of MBD2 on HDAC1/2.

Supplementary Material 7: Figure S4 The role of ABCB1 in chemoresistance of CCA.A-B: Overexpression of ABCB1 in TFK1 cells and RBE cells is validated by western blot. C: The IC50 of CCA cells to cisplatin upon MBD2 knockdown accompanied by ABCB1 overexpression. D: CCK-8 was used to detect the cells viability after treated with cisplatin. E: The representative image of liver in primary CCA mice models treated with cisplatin. Cis: cisplatin. F: HE staining of the liver in primary CCA mice models. G: The ratio of liver weight to body weight in primary CCA mice models. H: IHC staining of Ki-67 in liver of primary CCA mice models. The right panel is the statistical of Ki-67 rate. *P<0.05, **P<0.01, ***P<0.001.

Supplementary Material 8: Figure S5 MBD2 and WDR5 bind to the promoter of ABCB1 to promote its expression. A-D: The ChIP-qPCR was used to examine the binding of MBD2, WDR5 and H3K4me3 to ABCB1 in MBD2 or WDR5 overexpression RBE cells. Anti-Flag antibody was used for ChIP-qPCR to detect the enrichment of MBD2-Flag or WDR5-Flag in the binding region; Anti-H3K4me3 antibody was used for ChIP-qPCR to detect the enrichment of H3K4me3 in the binding region. **P<0.01, ***P<0.001.

Supplementary Material 9: Figure S6 The role of WDR5 and KMT2A in the prognosis of CCA.A-B: The mRNA expression level of WDR5 and KMT2A in paratumor tissues (n=40) and CCA tissues (n=40). C and D: Kaplan-Meier analysis of overall survival rate in low expression and high expression of WDR5 (C) and KMT2A (D) respectively. **P<0.01.

## Data Availability

The datasets used and/or analyzed during the current study are available from the corresponding author on reasonable request. All data have been submitted as supplemental material to editors.

## Abbreviations

MBD2: Methyl-CpG-binding Domain 2
WDR5: WD Repeat Domain 5
CCA: Cholangiocarcinoma
IHC: Immunohistochemistry
ABCB1: ATP-Binding Cassette Transporter B1
H3K4me3: Trimethylation of Lysine 4 Of Histone H3
KMT2A: Lysine Methyltransferase 2 A
NuRD: Nucleosome Remodeling Complex
ChIP-seq: Chromatin Immunoprecipitation Sequencing
MDR: Multidrug Resistance
FBS: Fetal Bovine Serum
CDS: Coding Sequence
IC50: Half Maximal Inhibitory Concentration
CCK-8: Cell Counting Kit-8
ChIP-qPCR: Real-Time Quantitative Polymerase Chain Reaction
qPCR: Quantitative Polymerase Chain Reaction
IF: Immunofluorescence
BSA: Bovine Serum Albumin
ROC: Receiver Operating Characteristic
AUC: Area Under The Curve
IHC: Immunohistochemistry
TCGA: The Cancer Genome Atlas
GEO: Gene Expression Omnibus
HE: Hematoxylin and Eosin
CpG: Cytosine-Phosphate-Guanine
IP: Immunoprecipitation
co-IP: co-Immunoprecipitation
HDAC1/2: Histone Deacetylase 1/2
Co-IP-MS: Co-Immunoprecipitation Mass Spectrometry

## References

[1] Brindley PJ, Bachini M, Ilyas SI, Khan SA, Loukas A, Sirica AE, et al. Cholangiocarcinoma Nat Reviews Disease Primers. 2021;7:65. 34504109 10.1038/s41572-021-00300-2 PMC9246479

[2] Florio AA, Ferlay J, Znaor A, Ruggieri D, Alvarez CS, Laversanne M, et al. Global trends in intrahepatic and extrahepatic cholangiocarcinoma incidence from 1993 to 2012. Cancer. 2020;126:2666–78. 32129902 10.1002/cncr.32803 PMC7323858

[3] Forner A, Vidili G, Rengo M, Bujanda L, Ponz-Sarvisé M, Lamarca A. Clinical presentation, diagnosis and staging of cholangiocarcinoma. Liver International: Official J Int Association Study Liver. 2019;39(Suppl 1):98–107. 10.1111/liv.14086 30831002

[4] Roth GS, Neuzillet C, Sarabi M, Edeline J, Malka D, Lièvre A. Cholangiocarcinoma: what are the options in all comers and how has the advent of molecular profiling opened the way to personalised medicine ? European journal of cancer (Oxford, England: 1990). 2023; 179: 1–14. 10.1016/j.ejca.2022.11.006 36463640

[5] Khan SA, Thomas HC, Davidson BR, Taylor-Robinson SD, Cholangiocarcinoma. Lancet (London England). 2005;366:1303–14. 16214602 10.1016/S0140-6736(05)67530-7

[6] Wood KH, Zhou Z. Emerging Molecular and Biological functions of MBD2, a reader of DNA methylation. Front Genet. 2016;7:93. 27303433 10.3389/fgene.2016.00093 PMC4880565

[7] Du Q, Luu PL, Stirzaker C, Clark SJ. Methyl-CpG-binding domain proteins: readers of the epigenome. Epigenomics. 2015;7:1051–73. 25927341 10.2217/epi.15.39

[8] Tan CP, Nakielny S. Control of the DNA methylation system component MBD2 by protein arginine methylation. Mol Cell Biol. 2006;26:7224–35. 16980624 10.1128/MCB.00473-06 PMC1592890

[9] Baubec T, Ivánek R, Lienert F, Schübeler D. Methylation-dependent and -independent genomic targeting principles of the MBD protein family. Cell. 2013;153:480–92. 23582333 10.1016/j.cell.2013.03.011

[10] Günther K, Rust M, Leers J, Boettger T, Scharfe M, Jarek M, et al. Differential roles for MBD2 and MBD3 at methylated CpG islands, active promoters and binding to exon sequences. Nucleic Acids Res. 2013;41:3010–21. 23361464 10.1093/nar/gkt035 PMC3597697

[11] Angrisano T, Lembo F, Pero R, Natale F, Fusco A, Avvedimento VE, et al. TACC3 mediates the association of MBD2 with histone acetyltransferases and relieves transcriptional repression of methylated promoters. Nucleic Acids Res. 2006;34:364–72. 16410616 10.1093/nar/gkj400 PMC1331987

[12] Zhou K, Zhou M, Cheng L, Chen X, Wang X, Chu Y, et al. Loss of MBD2 attenuates MLL-AF9-driven leukemogenesis by suppressing the leukemic cell cycle via CDKN1C. Oncogenesis. 2021;10:79. 34789717 10.1038/s41389-021-00366-3 PMC8599466

[13] Xie Y, Wang F, Yu J, Zhang J, Liu Y, Li M et al. Silencing of MBD2 and EZH2 inhibits the proliferation of colorectal carcinoma cells by rescuing the expression of SFRP. Oncol Rep. 2021;46(6):250. 10.3892/or.2021.8201 PMC8524315 34617573

[14] Zhu D, Osuka S, Zhang Z, Reichert ZR, Yang L, Kanemura Y, et al. BAI1 suppresses Medulloblastoma formation by protecting p53 from Mdm2-Mediated degradation. Cancer Cell. 2018;33:1004–e165. 29894688 10.1016/j.ccell.2018.05.006 PMC6002773

[15] Lu K, Tao H, Si X, Chen Q. The histone H3 lysine 4 presenter WDR5 as an oncogenic protein and Novel Epigenetic Target in Cancer. Front Oncol. 2018;8:502. 30488017 10.3389/fonc.2018.00502 PMC6246693

[16] Dou Y, Milne TA, Ruthenburg AJ, Lee S, Lee JW, Verdine GL, et al. Regulation of MLL1 H3K4 methyltransferase activity by its core components. Nat Struct Mol Biol. 2006;13:713–9. 16878130 10.1038/nsmb1128

[17] Xu J, Li L, Xiong J, denDekker A, Ye A, Karatas H, et al. MLL1 and MLL1 fusion proteins have distinct functions in regulating leukemic transcription program. Cell Discovery. 2016;2:16008. 27462455 10.1038/celldisc.2016.8 PMC4869169

[18] Yu X, Li D, Kottur J, Kim HS, Herring LE, Yu Y, et al. Discovery of Potent and selective WDR5 proteolysis targeting chimeras as potential therapeutics for pancreatic Cancer. J Med Chem. 2023;66:16168–86. 38019706 10.1021/acs.jmedchem.3c01521 PMC10872723

[19] Cai WL, Chen JF, Chen H, Wingrove E, Kurley SJ, Chan LH et al. Human WDR5 promotes breast cancer growth and metastasis via KMT2-independent translation regulation. eLife. 2022;11:e78163. 10.7554/eLife.78163 PMC9584608 36043466

[20] Sun Y, Bell JL, Carter D, Gherardi S, Poulos RC, Milazzo G, et al. WDR5 supports an N-Myc Transcriptional Complex that drives a protumorigenic gene expression signature in Neuroblastoma. Cancer Res. 2015;75:5143–54. 26471359 10.1158/0008-5472.CAN-15-0423

[21] Hao J, Huang J, Hua C, Zuo Y, Yu W, Wu X, et al. A novel TOX3-WDR5-ABCG2 signaling axis regulates the progression of colorectal cancer by accelerating stem-like traits and chemoresistance. PLoS Biol. 2023;21:e3002256. 37708089 10.1371/journal.pbio.3002256 PMC10501593

[22] Yin W, Xiang D, Wang T, Zhang Y, Pham CV, Zhou S, et al. The inhibition of ABCB1/MDR1 or ABCG2/BCRP enables doxorubicin to eliminate liver cancer stem cells. Sci Rep. 2021;11:10791. 34031441 10.1038/s41598-021-89931-9 PMC8144399

[23] Engle K, Kumar G. Cancer multidrug-resistance reversal by ABCB1 inhibition: a recent update. Eur J Med Chem. 2022;239:114542. 35751979 10.1016/j.ejmech.2022.114542

[24] Wu G, Wang Q, Wang D, Xiong F, Liu W, Chen J, et al. Targeting polycomb repressor complex 2-mediated bivalent promoter epigenetic silencing of secreted frizzled-related protein 1 inhibits cholangiocarcinoma progression. Clin Translational Med. 2023;13:e1502. 10.1002/ctm2.1502 PMC10696163 38050190

[25] Florea AM, Büsselberg D. Cisplatin as an anti-tumor drug: cellular mechanisms of activity, drug resistance and induced side effects. Cancers. 2011;3:1351–71. 24212665 10.3390/cancers3011351 PMC3756417

[26] Valle J, Wasan H, Palmer DH, Cunningham D, Anthoney A, Maraveyas A, et al. Cisplatin plus gemcitabine versus gemcitabine for biliary tract cancer. N Engl J Med. 2010;362:1273–81. 20375404 10.1056/NEJMoa0908721

[27] Desai MA, Webb HD, Sinanan LM, Scarsdale JN, Walavalkar NM, Ginder GD, et al. An intrinsically disordered region of methyl-CpG binding domain protein 2 (MBD2) recruits the histone deacetylase core of the NuRD complex. Nucleic Acids Res. 2015;43:3100–13. 25753662 10.1093/nar/gkv168 PMC4381075

[28] Torchy MP, Hamiche A, Klaholz BP. Structure and function insights into the NuRD chromatin remodeling complex. Cell Mol Life Sci. 2015;72:2491–507. 25796366 10.1007/s00018-015-1880-8 PMC11114056

[29] Wang Z, Zang C, Cui K, Schones DE, Barski A, Peng W, et al. Genome-wide mapping of HATs and HDACs reveals distinct functions in active and inactive genes. Cell. 2009;138:1019–31. 19698979 10.1016/j.cell.2009.06.049 PMC2750862

[30] Wu CP, Hsieh YJ, Tseng HY, Huang YH, Li YQ, Hung TH, et al. The WD repeat-containing protein 5 (WDR5) antagonist WDR5-0103 restores the efficacy of cytotoxic drugs in multidrug-resistant cancer cells overexpressing ABCB1 or ABCG2. Volume 154. Biomedicine & pharmacotherapy = Biomedecine & pharmacotherapie; 2022. p. 113663. 10.1016/j.biopha.2022.113663 36081287

[31] Karatas H, Townsend EC, Cao F, Chen Y, Bernard D, Liu L, et al. High-affinity, small-molecule peptidomimetic inhibitors of MLL1/WDR5 protein-protein interaction. J Am Chem Soc. 2013;135:669–82. 23210835 10.1021/ja306028q PMC5180416

[32] Ng HH, Zhang Y, Hendrich B, Johnson CA, Turner BM, Erdjument-Bromage H, et al. MBD2 is a transcriptional repressor belonging to the MeCP1 histone deacetylase complex. Nat Genet. 1999;23:58–61. 10471499 10.1038/12659

[33] Ai K, Pan J, Zhang P, Li H, He Z, Zhang H, et al. Methyl-CpG-binding domain protein 2 contributes to renal fibrosis through promoting polarized M1 macrophages. Cell Death Dis. 2022;13:125. 35136032 10.1038/s41419-022-04577-3 PMC8826408

[34] Mahmood N, Arakelian A, Szyf M, Rabbani SA. Methyl-CpG binding domain protein 2 (Mbd2) drives breast cancer progression through the modulation of epithelial-to-mesenchymal transition. Experimental & molecular medicine; 2024. 10.1038/s12276-024-01205-2 PMC11058268 38556549

[35] Liu Z, Sun L, Cai Y, Shen S, Zhang T, Wang N, et al. Hypoxia-Induced suppression of alternative splicing of MBD2 promotes breast Cancer metastasis via activation of FZD1. Cancer Res. 2021;81:1265–78. 33402389 10.1158/0008-5472.CAN-20-2876

[36] Stirzaker C, Song JZ, Ng W, Du Q, Armstrong NJ, Locke WJ, et al. Methyl-CpG-binding protein MBD2 plays a key role in maintenance and spread of DNA methylation at CpG islands and shores in cancer. Oncogene. 2017;36:1328–38. 27593931 10.1038/onc.2016.297

[37] Martin V, Jørgensen HF, Chaubert AS, Berger J, Barr H, Shaw P, et al. MBD2-mediated transcriptional repression of the p14ARF tumor suppressor gene in human colon cancer cells. Pathobiol J ImmunoPathol Mol Cell Biol. 2008;75:281–7. 10.1159/000151708 18931530

[38] Shang S, Li X, Azzo A, Truong T, Dozmorov M, Lyons C, et al. MBD2a-NuRD binds to the methylated γ-globin gene promoter and uniquely forms a complex required for silencing of HbF expression. Proc Natl Acad Sci USA. 2023;120:e2302254120. 37307480 10.1073/pnas.2302254120 PMC10288633

[39] Zhang L, Wang S, Wu GR, Yue H, Dong R, Zhang S, et al. MBD2 facilitates tumor metastasis by mitigating DDB2 expression. Cell Death Dis. 2023;14:303. 37142578 10.1038/s41419-023-05804-1 PMC10160113

[40] Wang Y, Zhang L, Huang T, Wu GR, Zhou Q, Wang FX et al. The methyl-CpG-binding domain 2 facilitates pulmonary fibrosis by orchestrating fibroblast to myofibroblast differentiation. Eur Respir J. 2022;60(3):2003697. 10.1183/13993003.03697-2020 PMC9520034 35086828

[41] Bochyńska A, Lüscher-Firzlaff J, Lüscher B. Modes of Interaction of KMT2 Histone H3 Lysine 4 Methyltransferase/COMPASS Complexes with Chromatin. Cells. 2018; 7. 10.3390/cells7030017 PMC5870349 29498679

[42] Dharmarajan V, Lee JH, Patel A, Skalnik DG, Cosgrove MS. Structural basis for WDR5 interaction (Win) motif recognition in human SET1 family histone methyltransferases. J Biol Chem. 2012;287:27275–89. 22665483 10.1074/jbc.M112.364125 PMC3431640

[43] Soares LM, He PC, Chun Y, Suh H, Kim T, Buratowski S. Determinants of histone H3K4 methylation patterns. Mol Cell. 2017;68(4):773–785.e6. 10.1016/j.molcel.2017.10.013 PMC5706784 29129639

[44] Thomas LR, Wang Q, Grieb BC, Phan J, Foshage AM, Sun Q, et al. Interaction with WDR5 promotes target gene recognition and tumorigenesis by MYC. Mol Cell. 2015;58:440–52. 25818646 10.1016/j.molcel.2015.02.028 PMC4427524

[45] Carugo A, Genovese G, Seth S, Nezi L, Rose JL, Bossi D, et al. In vivo functional platform targeting patient-derived xenografts identifies WDR5-Myc Association as a critical determinant of pancreatic Cancer. Cell Rep. 2016;16:133–47. 27320920 10.1016/j.celrep.2016.05.063

