# Supplementary figures and images for: MBD2 regulates the progression and chemoresistance of cholangiocarcinoma through interaction with WDR5
Source: J Exp Clin Cancer Res. 2024 Sep 30;43:272. doi: 10.1186/s13046-024-03188-4 (PMC11440836; doi:10.1186/s13046-024-03188-4)

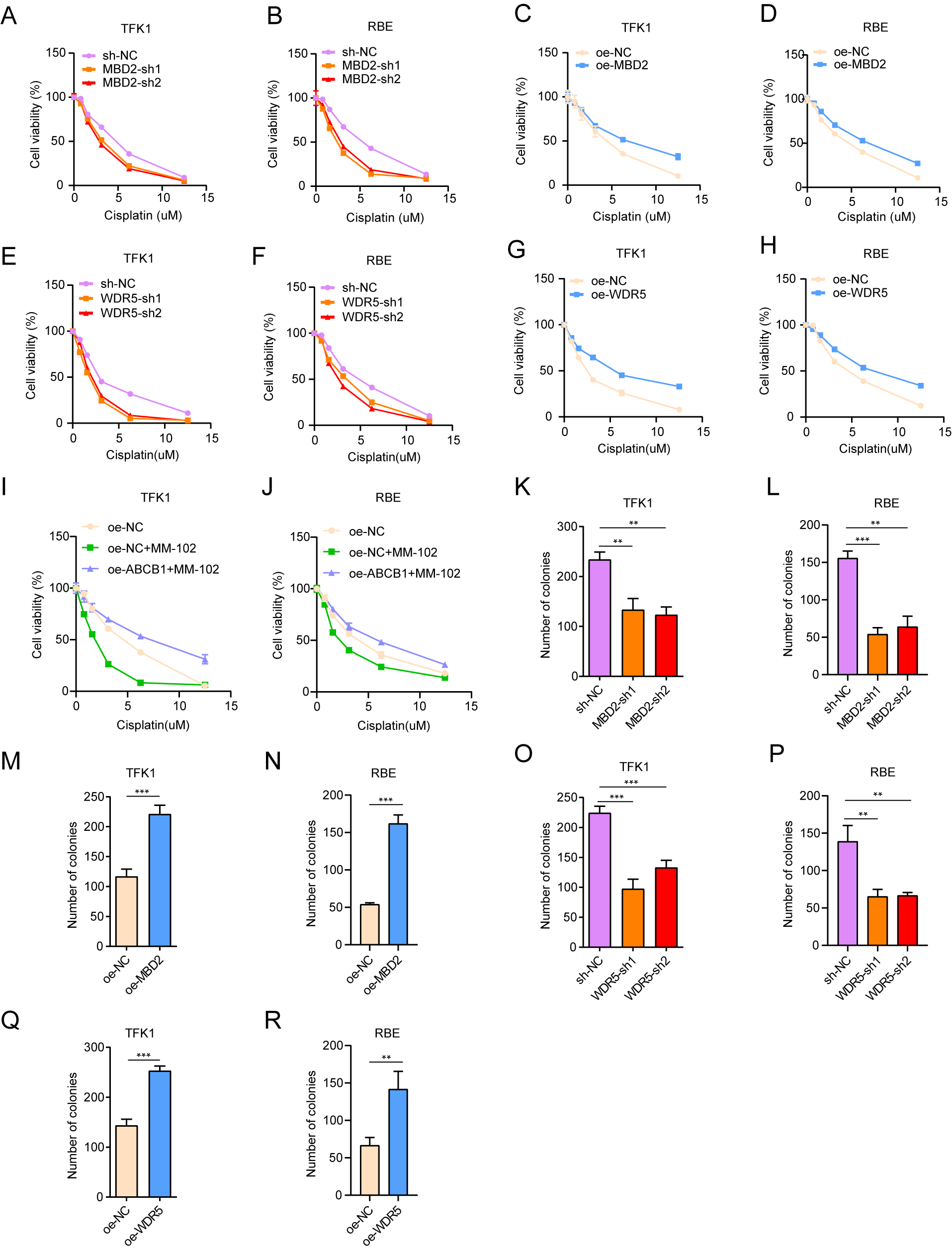

Supplement: Supplementary file 4 — Supplementary Material 4: Figure S1 The viability of CCA cells to cisplatin and the statistical chart of plate clones. A-D: TFK1 and RBE cells knocking down or overexpressing MBD2 were treated with cisplatin, CCK-8 was used to detect the cells viability. E-H: TFK1 and RBE cells knocking down or overexpressing WDR5 were treated with cisplatin, CCK-8 was used to detect the cells viability. I-J: TFK1 and RBE cells were treated with MM-102 and cisplatin, CCK-8 was used to detect the cells viability. K-N: The statistical volume of plate cloning assay in MBD2 knockdown or overexpression cells. O-R: The statistical volume of plate cloning assay in WDR5 knockdown or overexpression cells. **P<0.01, ***P<0.001. [file 13046_2024_3188_MOESM4_ESM.tif]

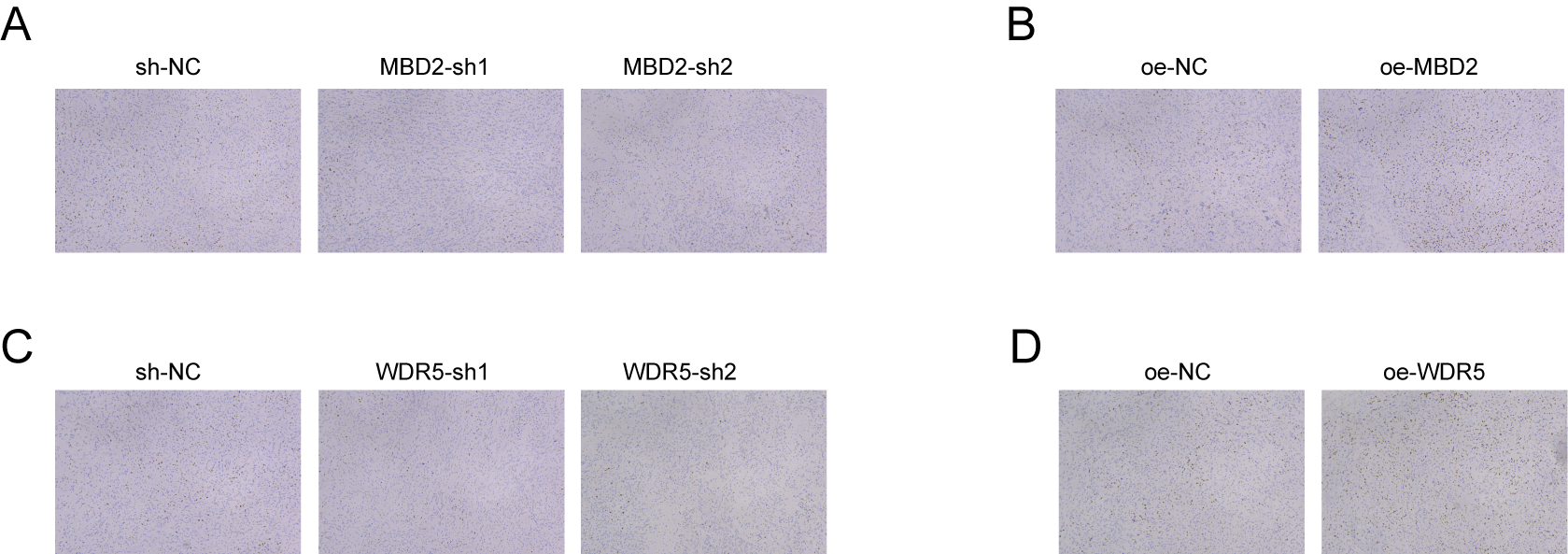

Supplement: Supplementary file 5 — Supplementary Material 5: Figure S2 The IHC of tumor from xenograft mice models. A-B: The IHC staining of Ki-67 in MBD2 knockdown and overexpression xenograft tumor derived from TFK1 cells. C-D: The IHC staining of Ki-67 in WDR5 knockdown and overexpression xenograft tumor derived from TFK1cells. [file 13046_2024_3188_MOESM5_ESM.tif]

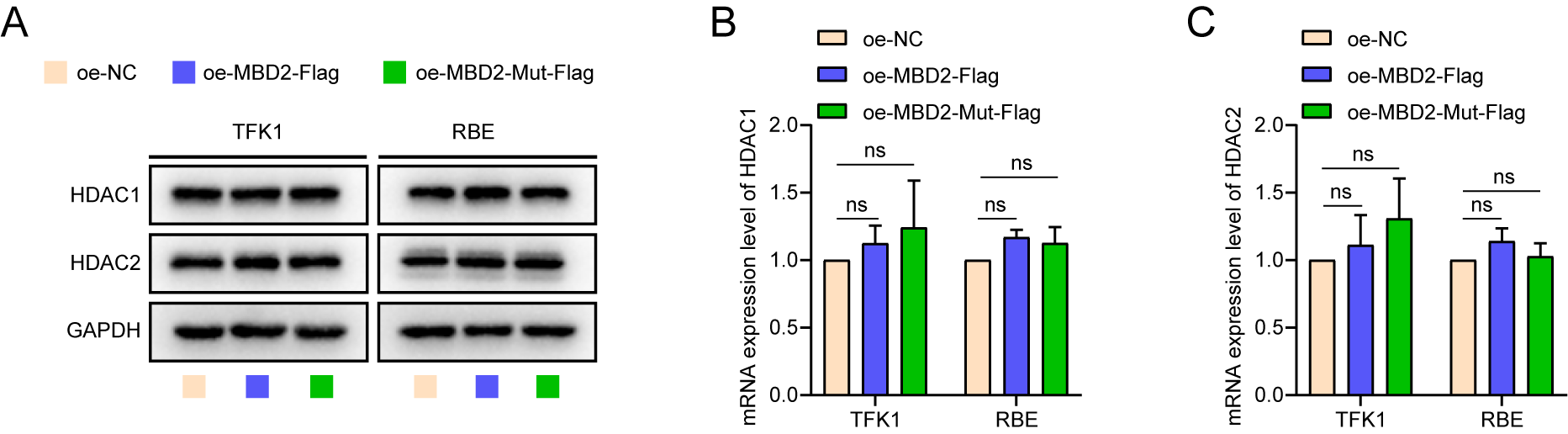

Supplement: Supplementary file 6 — Supplementary Material 6: Figure S3 MBD2 does not affect the expression of HDAC1/2A-C: Western blot (A) and RT-PCR (B and C) were used to detect the effect of MBD2 on HDAC1/2. [file 13046_2024_3188_MOESM6_ESM.tif]

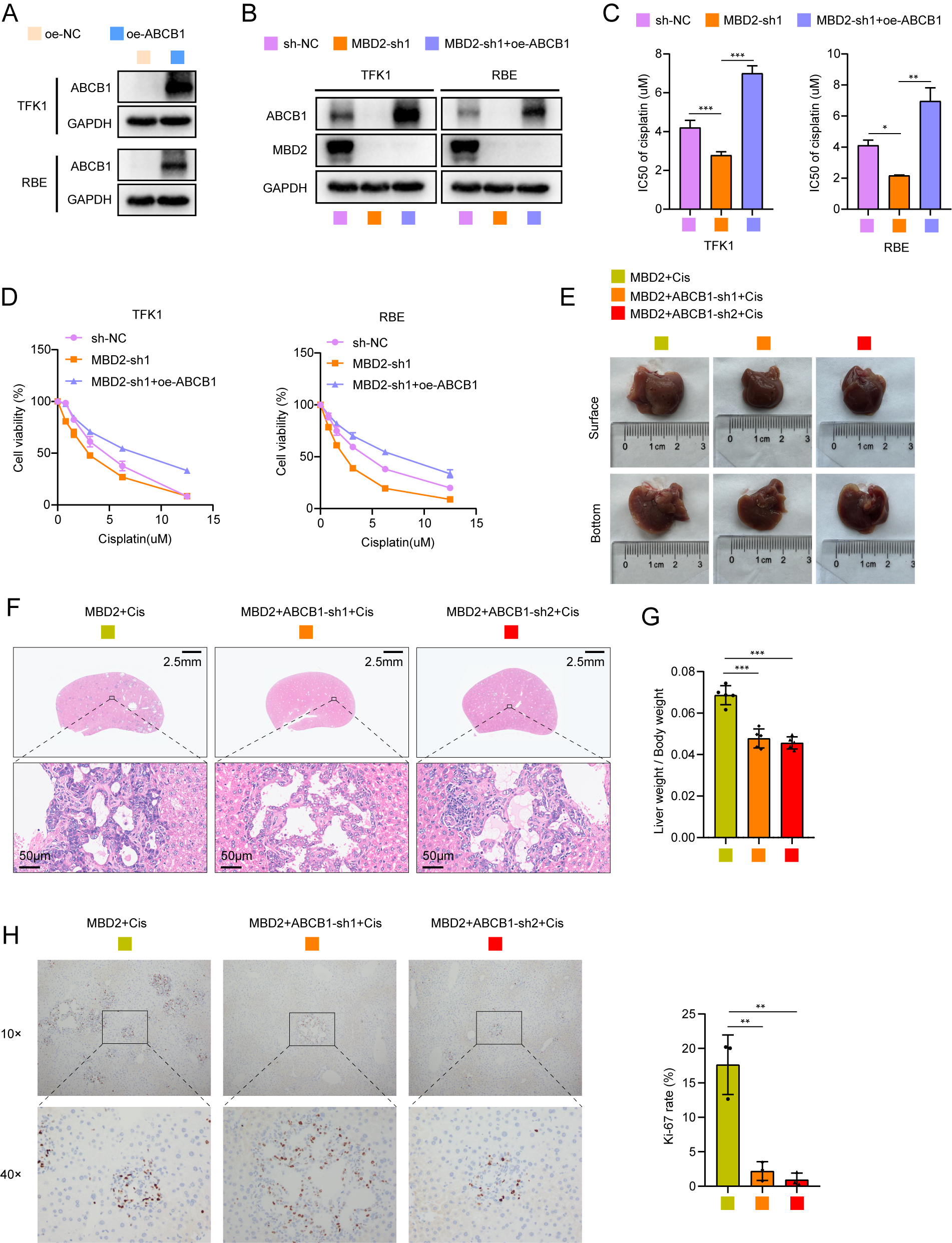

Supplement: Supplementary file 7 — Supplementary Material 7: Figure S4 The role of ABCB1 in chemoresistance of CCA.A-B: Overexpression of ABCB1 in TFK1 cells and RBE cells is validated by western blot. C: The IC50 of CCA cells to cisplatin upon MBD2 knockdown accompanied by ABCB1 overexpression. D: CCK-8 was used to detect the cells viability after treated with cisplatin. E: The representative image of liver in primary CCA mice models treated with cisplatin. Cis: cisplatin. F: HE staining of the liver in primary CCA mice models. G: The ratio of liver weight to body weight in primary CCA mice models. H: IHC staining of Ki-67 in liver of primary CCA mice models. The right panel is the statistical of Ki-67 rate. *P<0.05, **P<0.01, ***P<0.001. [file 13046_2024_3188_MOESM7_ESM.tif]

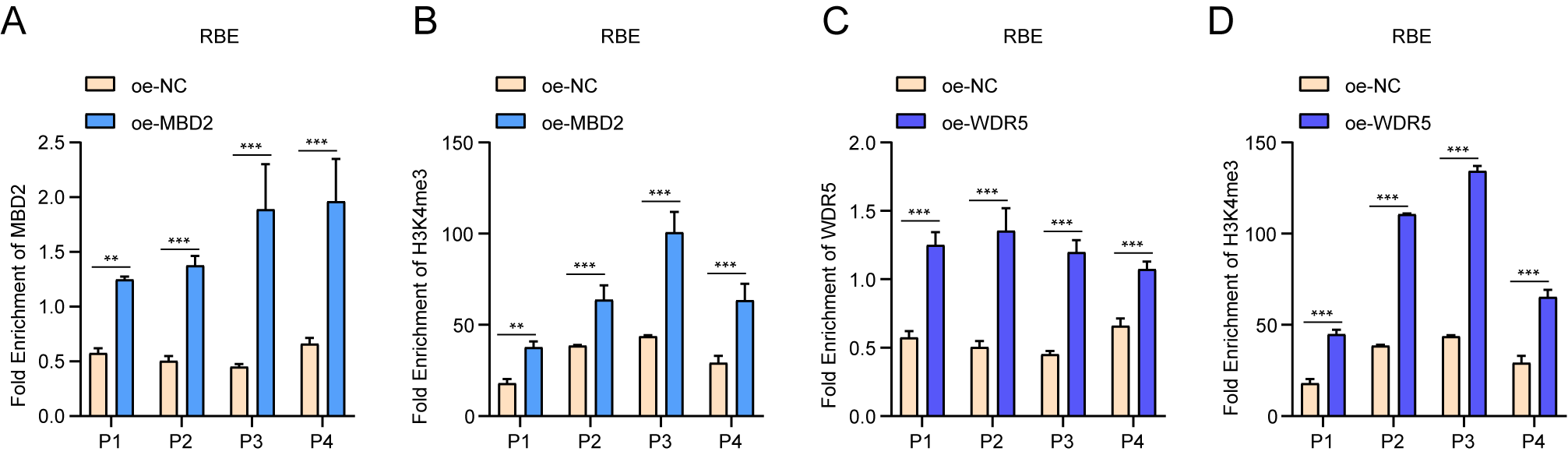

Supplement: Supplementary file 8 — Supplementary Material 8: Figure S5 MBD2 and WDR5 bind to the promoter of ABCB1 to promote its expression. A-D: The ChIP-qPCR was used to examine the binding of MBD2, WDR5 and H3K4me3 to ABCB1 in MBD2 or WDR5 overexpression RBE cells. Anti-Flag antibody was used for ChIP-qPCR to detect the enrichment of MBD2-Flag or WDR5-Flag in the binding region; Anti-H3K4me3 antibody was used for ChIP-qPCR to detect the enrichment of H3K4me3 in the binding region. **P<0.01, ***P<0.001. [file 13046_2024_3188_MOESM8_ESM.tif]

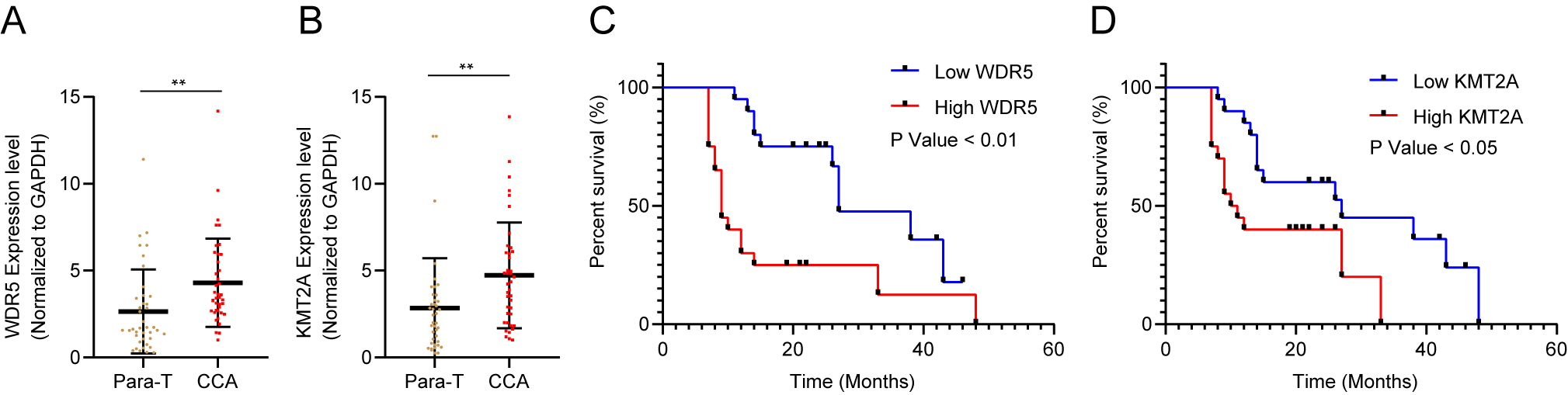

Supplement: Supplementary file 9 — Supplementary Material 9: Figure S6 The role of WDR5 and KMT2A in the prognosis of CCA.A-B: The mRNA expression level of WDR5 and KMT2A in paratumor tissues (n=40) and CCA tissues (n=40). C and D: Kaplan-Meier analysis of overall survival rate in low expression and high expression of WDR5 (C) and KMT2A (D) respectively. **P<0.01. [file 13046_2024_3188_MOESM9_ESM.tif]
